# Exploring the Biocompatibility and Therapeutic Potential of Graphene Oxide-Based Nanomaterials in Modern Medicine. A Comprehensive Review

**DOI:** 10.1007/s12010-026-05828-1

**Published:** 2026-08-04

**Authors:** Fozia Aslam, Salamat Ullah Khan Niazi, Muhammad Azam Qamar, Aman Khalid, Muhammad Nouman, Muhammad Zahid, Zulfiqar Ahmad Rehan, Ahmad Farhan

**Affiliations:** 1 Center for Biomedical Optics and Photonics (CBOP) & College of Physics and Optoelectronic Engineering, Shenzhen University, Shenzhen, China; 2 Institute of Advanced Study in Nuclear Energy & Safety, College of Physics and Optoelectronics Engineering, Shenzhen University, Shenzhen, China; 3 Department of Chemistry, School of Science, University of Management and Technology, Lahore, 54700 Pakistan; 4 Department of Chemistry, University of Agriculture Faisalabad, Faisalabad, Punjab 38040 Pakistan; 5 International Center for Interdisciplinary Research in Sciences, The University of Lahore, Lahore, Pakistan; 6 College of Chemistry and Environmental Engineering, Shenzhen University, Shenzhen, 518060 People’s Republic of China; 7 Department of Chemistry, College of Science, Sultan Qaboos University, Al- Khoud-123, Muscat, Oman

**Keywords:** Graphene, Drug delivery systems, Biomedical applications, Bioimaging

## Abstract

Graphene oxide, a 2D nanomaterial, has attracted significant attention for biomedical applications due to its exceptional properties, including tunable surface chemistry, excellent dispersibility, and abundant oxygen-containing functional groups that facilitate facile modification. Recent advancements in the synthesis and functionalization of graphene oxide-based materials have addressed their limitations, such as poor solubility and cytotoxicity, thereby making them safer and more effective for biomedical applications. This paper thoroughly outlines the structural attributes and principal qualities of graphene oxide-based materials, highlighting their mechanical strength, thermal stability, impermeability, electrical conductivity, and biological properties. Emerging developments in graphene oxide-based nanocomposites for fluorescence imaging, magnetic resonance imaging, photoacoustic imaging, Raman spectroscopy imaging, and multifunctional therapeutic platforms are thoroughly examined. Their effectiveness in biomedical scaffolds, wound-healing systems, controlled drug-release platforms, and antimicrobial coatings has been highlighted. Notwithstanding considerable advancements, issues related to cytotoxicity, biodegradability, long-term biosafety, and scalable production continue to impede broad clinical translation. This study offers a cutting-edge overview of graphene oxide-based biomedical systems and outlines promising pathways for advancing safer, more effective, and clinically relevant graphene-based healthcare technologies.

## Introduction

In modern medicine, the treatment of complex diseases such as cancer, neurodegenerative disorders [1], and chronic wounds [2] presents significant challenges. Traditional therapies often suffer from issues such as non-specific drug delivery, high toxicity, and poor targeting of affected cells [3]. As a result, there is an increasing need for innovative therapeutic strategies that can overcome these limitations and provide more effective, targeted treatments. Globally, chronic wounds, including diabetic ulcers and venous ulcers, have become more prevalent with the aging population and rising incidences of chronic diseases like diabetes and vascular diseases. For example, the prevalence of chronic leg ulcers is estimated at 1.51 per 1000 population [4]. Similarly, chronic wounds, particularly pressure ulcers, are notably more common in elderly populations, with the incidence increasing significantly with age [5]. These wounds not only result in considerable medical expenses but also cause prolonged suffering and loss of function, contributing to a reduced standard of living.

The graphene oxide (GO) family of materials has recently garnered significant interest due to its wide range of potential applications in the biomedical industry [6]. Due to its two-dimensional (2D) carbon structure, graphene oxide (GO) exhibits exceptional mechanical strength, a large surface area, and functional groups that facilitate surface modification [7, 8]. The remarkable electron-transport properties and extensive interfacial contact area of graphene oxide substantially improve photocatalytic performance by facilitating charge-carrier separation and minimizing recombination losses in hybrid nanocomposites [9–11]. Graphene oxide has been used in different fields of biomedicine, such as drug transport, bioimaging, tissue engineering, and cancer treatment [12]. The biomedical potential of GO has been largely driven by its remarkable versatility. It may be modified to produce biocompatible composites for targeted drug delivery and gene therapy, given the abundance of oxygen-containing groups, such as hydroxyl, carboxyl, and epoxy, on its surface. According to research, GO may be used as a vehicle for many medicinal substances, allowing for regulated release while reducing the negative effects of traditional pharmaceuticals [13]. Targeting molecules can be attached to functionalized GO, thereby improving its specificity for diseased cells, especially in cancer therapies. Furthermore, it is used in bioimaging as a contrast agent in magnetic resonance imaging [14] and fluorescence imaging [15].

Synthesis and functionalization of GO have recently made significant advancements in addressing some of the challenges associated with its biomedical applications. Traditionally, poor solubility and potential cytotoxicity have limited its use. However, recent research has led to the development of safer and more effective methods to enhance its water solubility and biocompatibility. Non-toxic reducing agents have been explored to produce reduced graphene oxide (rGO), which exhibits improved dispersibility in aqueous environments while maintaining its desirable properties for drug delivery and imaging applications [16]. The biodegradability of GO is also a critical factor in its long-term medical applications. Recent studies have shown that GO can degrade in the human body via specific enzymes, providing a safer alternative to other nanomaterials that may accumulate in tissues [17]. This biocompatibility and degradability make GO a promising candidate for a variety of clinical applications, including wound healing and the treatment of neurodegenerative diseases like Alzheimer’s, where GO nanocarriers can help reduce amyloid-beta toxicity [18].

The reviews that do cover graphene oxide (GO) functionalization in the context of biomedical application obstacles do so in a limited scope. Most reviews on GO cover the basic scientific properties, methods, and broad applications in drug delivery, tissues engineering, and other areas in biomedical engineering. However, most of these reviews ignore GO’s toxicity, biocompatibility, and possible controlled degradation in biological systems. While these reviews speak of GO’s great potential, they fail to recognize GO’s limitations in clinical applications, notably safety and efficacy. This paper attempts to address and focus on GO functionalization to address biocompatibility and limit potential toxicity. While there is literature available in the theoretical aspect of GO, the current paper will discuss the possible and practical aspects of GO. Further, new theoretical concepts to limit immunological response and accumulation of GO in tissues will be discussed. This research will focus on the safe utilization of GO and will focus on the available clinical applications for the treatment of chronic wounds, cancer, and other complicated pathologies. Unlike other research papers that consider GO to be a universal solution, this research will consider the limitations of GO in various biomedical applications and will also address the issues of surface modification and GO functionalization to limit toxicity.

Our review offers a broad, multi-domain overview of GO-based nanomaterials across modern medicine (bioimaging, drug/gene delivery, tissue engineering, wound healing, antimicrobial coatings, and biosensing), whereas the chapter is narrowly focused on cancer theranostics (imaging + PTT/PDT/drug delivery in oncology) [19]. We provide deeper, updated analysis of biocompatibility, enzymatic biodegradability, functionalization strategies for toxicity reduction, and recent first-in-human/large-animal safety data, with dedicated emphasis on long-term implantation risks.

## Structure of Graphene Oxide

Carbon-based nanomaterials, especially graphene and its derivatives, have emerged as promising substances due to their exceptional structural, electrical, and thermal properties, facilitating various applications in biomedicine [20]. Extensive experimental investigations of the GO structure have been carried out over the years, utilizing various methods such as ultra-high-resolution transmission electron microscopy, X-ray diffraction, ^13^C and ^1^H nuclear magnetic resonance, and annular dark-field imaging [21–24]. Multiple efforts to explain the structure of graphene oxide have yielded competing structural models. The primary reasons for this are the complexity of the material and the uniqueness of each sample resulting from its variable stoichiometry. A combination of sp^2^ and sp^3^ hybridized carbon atoms is produced when graphene oxide monolayer sheets of graphite are functionalized with hydroxyl, carboxyl, and epoxy oxygen groups on both the basal plane and the edges [25]. Numerous GO models have been developed based on various studies and theoretical calculations. In their first proposal from 1939, Hofmann and Rudolf envisioned a graphite monolayer with many epoxy groups distributed at random [26]. In 1946, Ruess modified the structural model of graphene oxide by incorporating hydroxyl groups and suggesting a partial transformation of sp^2^-hybridized carbon atoms into sp^3^-hybridized carbon atoms [27]. Subsequently, Scholz and Boehm proposed a less-organized structure, lacking ether oxygen groups, characterized by undulating carbon layers that incorporate hydroxyl and carbonyl functionalities, as well as repeating C–C single bonds [28]. Additionally, in 1994, Nakajima and Matsuo developed a model that closely resembled a graphite intercalation compound [29]. The LK model proposed by Lerf and Klinowski consists of two distinct regions: one containing six-membered aliphatic rings and the other containing nonoxidized aromatic benzene rings [22].

The extent to which the material has been oxidized determines the relative sizes of the two zones. Adorned with epoxide groups and double bonds, the model is mostly composed of aromatic molecules. The hydroxyl groups bound to carbon atoms produce a slightly warped tetrahedral arrangement that leads to the wrinkling of the monolayer. Both the top and bottom carbon monolayers have oxygen functional groups attached, forming two layers of oxygen atoms with different proportions of epoxide and hydroxyl groups, closely packed together. Across the carbon monolayer, aromatic compounds and oxidized rings are present. The acidity of GO is caused by the presence of hydroxyl and carboxyl groups at the lattice surfaces. The LK model is a widely recognized representation of graphene oxide with a moderate oxidation state. Graphene oxide and its derivatives have become the focus of researchers worldwide since their discovery. The 2006 model by Szábó and Dékány, derived from an analysis of prior models, excluded carboxylic acids and comprised two principal components: cyclohexane chairs linked by 1,3-epoxide and intermediate alcohols, and corrugated hexane ribbons interspersed with quinones and ketones. Unlike the static LK model, the dynamical structural model proposed by Dimiev, Alemany, and Tour in 2013 [30] described the formation of different carbon structures containing bound water. Liu et al. recently provided experimental proof of C = O bonding at the carbon monolayer edge, which serves as the foundation for prior theories, notably the LK model [31, 32]. Graphene oxide comprises topological defects such as pentagons, heptagons, and octagons, as well as structural faults comprising adatoms, vacancies, and adsorbed pollutants.

**Fig. 1:**
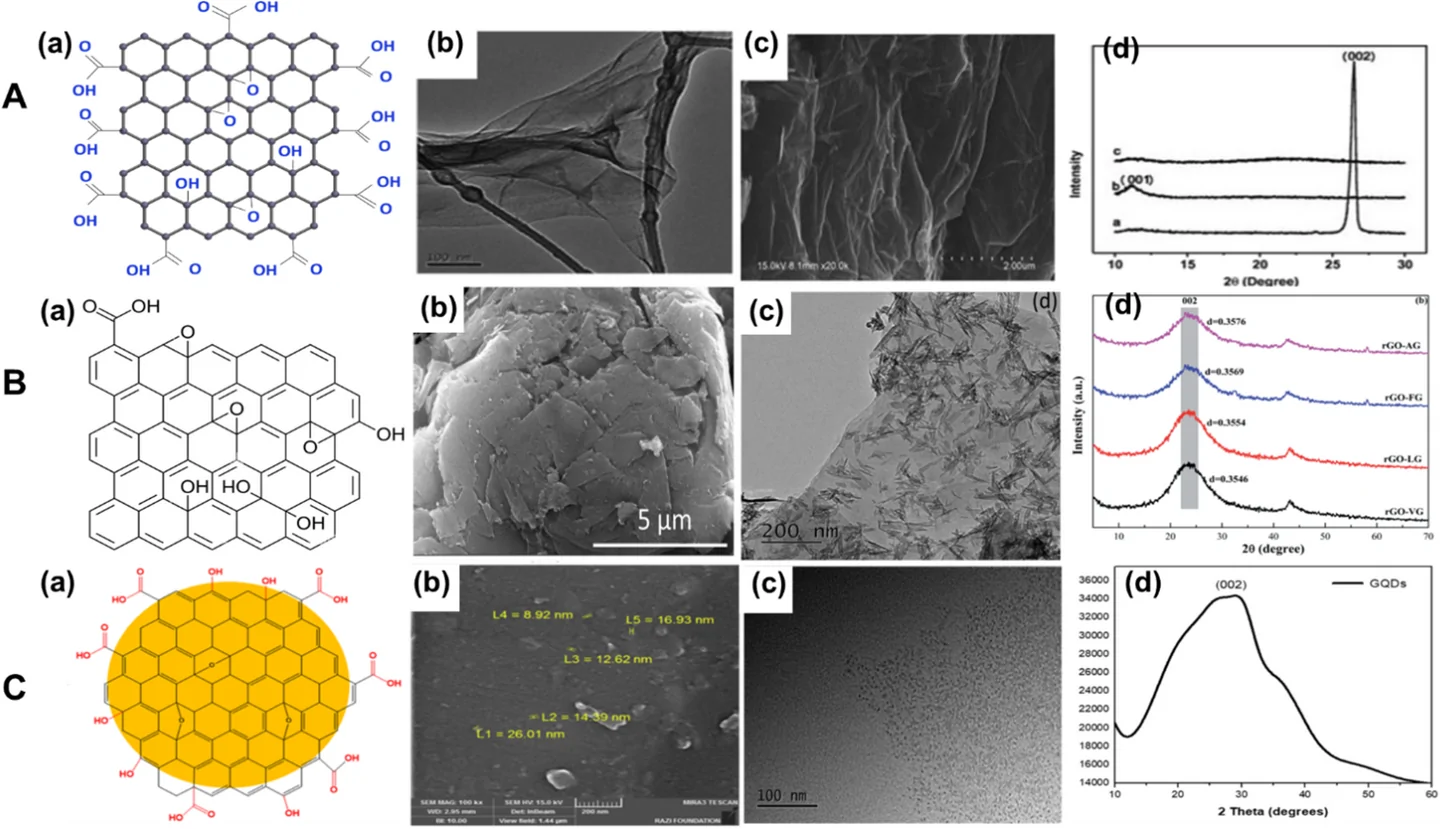
**(A) (a-d)** Structure, SEM, TEM, and XRD of GO. Reproduced with permission from [42] **(B) (a-d)** Structure, SEM, TEM, and XRD image of rGO. Reproduced with permission from [43, 44] **(C) (a-d)** Structure, SEM, TEM, XRD image of GOQDs. Reproduced with permission from [45, 46]

Moreover, oxidation in the presence of concentrated acids facilitates the generation of oxidation byproducts, comprising low-molecular-weight oxidized carbon particles [33, 34]. Various oxidation byproducts, including highly oxidized polyaromatic particles and less oxidized GO platelets, are attached due to hydrogen bonding, π-π stacking, and van der Waals interactions [35]. Longer reactions of graphite with concentrated acids result in more oxidation debris [36, 37]. These fragments cannot be removed without first doing a base wash [37–39]. In terms of oxidation level, pure GO, which does not include any oxidation residues, is on par with chemically reduced GO [40]. Kim et al. reported a two-step thermal method for the synthesis of hierarchically porous graphene. Initially, graphene oxide (GO) underwent annealing at 650 °C in an atmospheric environment, resulting in the decomposition of oxygen functionalities that emitted CO_2_/CO and expanded the sheets into slit nanopores, producing activated reduced graphene oxide (ArGO). A subsequent annealing at 600 °C in nitrogen eliminated residual oxygen groups and produced more micropores, yielding thermally treated ArGO [41]. Structure, SEM, TEM, and XRD images of graphene oxide (GO), reduced graphene oxide (rGO), and graphene oxide quantum dots (GOQDs) are shown in Fig. 1.

## Methods for Graphene Oxide Synthesis

**Fig. 2:**
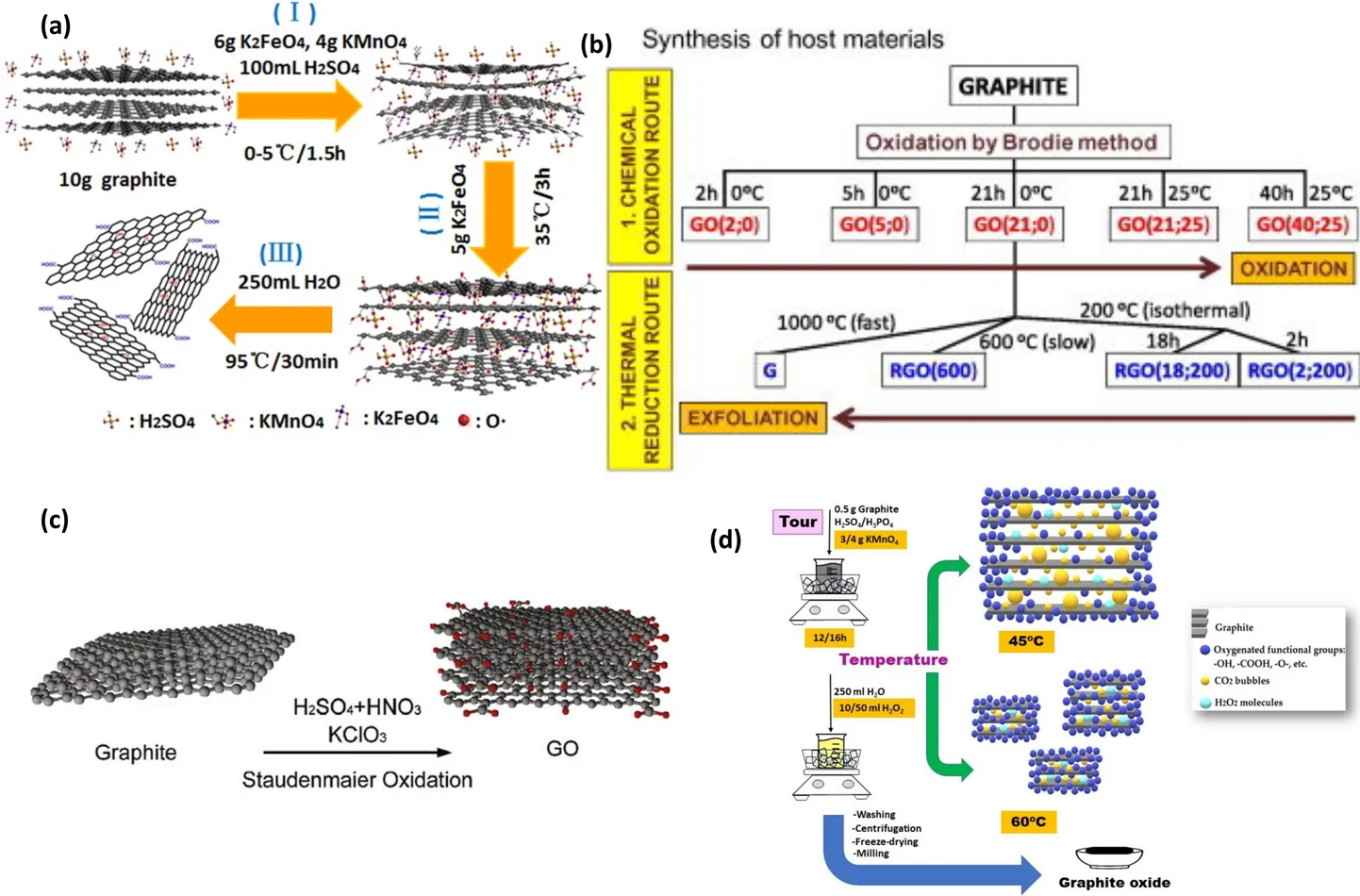
**(a)** Synthetic routes of Graphene oxide through the Modified Hummers Method. Produced under common creative license 4.0 [50]., **(b)** Brodie method. Produced under common creative license CC-BY [53], **(c)** Staudenmaier Oxidation. Reproduced with permission from [54], **(d)** Produced under common creative license 4.0. Tour Method [55]

Among the several permanganate techniques developed by Hummers and Offeman, the most commonly used and effective in the past is the Hummers method, as shown in Fig. 2(a). Producing graphene oxide with this method is a rather quick example of classical technology [47]. A small amount of sodium nitrate, sulfuric acid, and potassium permanganate is the component of the reaction mixture. Between eight and twelve hours is the typical response time. Because it does not involve the creation of explosive ClO_2_, this method is much safer. After everything is finished, a diluted H_2_O_2_ solution is used to neutralize the excess potassium permanganate. The carbon-to-oxygen ratios of BR-GO (2.2) and Hummers’ graphene oxide (2.25) are quite comparable. The method’s production of nitrogen oxides makes it harmful to the environment. Low- and room-temperature methods, as well as nitrate-free two-step and co-oxidant variations, are among the many modified Hummers’ processes available [48–52].

In 1859, while investigating the reactivity of flake graphite, British scientist B. C. Brodie synthesized graphite oxide for the first time, as shown in Fig. 2 (b) [56, 57]. The oxidizing agent used in this chlorate route is potassium chlorate. Benjamin Brodie used a wide variety of strong oxidizing chemicals to finally uncover graphite’s structure. In the experiment, Brodie heated graphite with a mixture of fuming nitric acid and potassium chlorate at 60 °C for 4 days to produce BR-GO, or graphene oxide. His extensive series of oxidative treatments (4–7) yielded a C_11_H_4_O_5_ formula, corresponding to a C/O ratio of 2.2, according to his calculations [57]. Product solubility in pure water was confirmed; nevertheless, flocculation in an acidic environment was observed. Brodie called it “graphic acid” since it didn’t react much with litmus paper. One such way to make chlorate is via the Staudenmaier process, as shown in Fig. 2 (c) [58]. In order to alter the chlorate addition method, Staudenmaier used sulfuric acid in the mixture. The risk of heat production and the formation of explosive by-products was reduced by gradually adding potassium chlorate to the mixture. The reaction time was shortened due to the increased acidity of the air [59]. The oxidation level of graphene oxide increases as the concentration of nitric acid decreases.

## Advanced Methods of Graphene Oxide Synthesis

It is possible to produce graphite oxide and graphene oxide using a variety of ways. The standard procedure involves exposing the material to an acidic atmosphere and then oxidizing it. Two further possibilities are microbiological and electrochemical approaches. Tour’s graphene oxide process is classified as a permanganate method shown in Fig. 2 (d) [60]. In an ice bath, a 6:1 ratio of graphite to potassium permanganate is introduced, while a 1:9 ratio of phosphoric acid to sulfuric acid is combined. Stir the mixture for 12 h after heating it to 50 °C. After the mixture has cooled, it is spread over the ice. To remove the excess potassium permanganate, 30% H_2_O_2_ is then added. Phosphoric acid acts as an etching agent, a dispersant, and an oxidation stabilizer, ensuring the safe synthesis of graphene oxide. This approach yields graphene oxide with a greater oxidation level and a more uniform structure.

Modern methods for oxidizing graphite to GO include chlorate and permanganate procedures, but also employ potassium chromate using nitric or perchloric acid, or to do the experiment using Jones’s conditions [61, 62]. Another, less dangerous option for making graphene oxide is to combine potassium ferrate with sulfuric acid [63]. While these results are encouraging, another study shows that GO synthesized using potassium ferrate is impossible [64]. Also, at 50 °C using Fe(VI) as a catalyst, graphite may be oxidized in water with H_2_O_2_ with benzoyl peroxide at 110 °C [65, 66]. There is little change in the final product when the reaction time is cut in half, from 12 h to 1, according to Jankovský et al., who modified Tour’s technique [67]. Electrochemical synthesis provides an alternate way to chemical techniques for synthesizing graphene oxide, which could be crucial for industrial-scale production. Due to its reduced equipment cleaning requirements and ability to reuse the electrolyte, electrochemical manufacturing is preferable to chemical production from an environmental sustainability standpoint [68, 69]. One possible explanation for electrochemical graphene oxide’s (EGO) higher quality than traditional techniques is that it uses aqueous electrolytes and doesn’t employ oxidizing agents, which eliminates impurities [69]. It is also possible to control oxidation levels and fault density because of the variety of experimental setups.

## Properties of Graphene Oxide-Based Composites

Graphene-based materials demonstrate distinctive physical, chemical, and biological properties, making them attractive for various applications. Their performance is closely associated with vital characteristics, including thermal stability, impermeability, mechanical strength, biocompatibility, and electrical conductivity, that are examined in this section. A comprehensive grasp of these features establishes the basis for assessing their suitability for use in biomedical technologies [70].

### Mechanical Properties

**Fig. 3:**
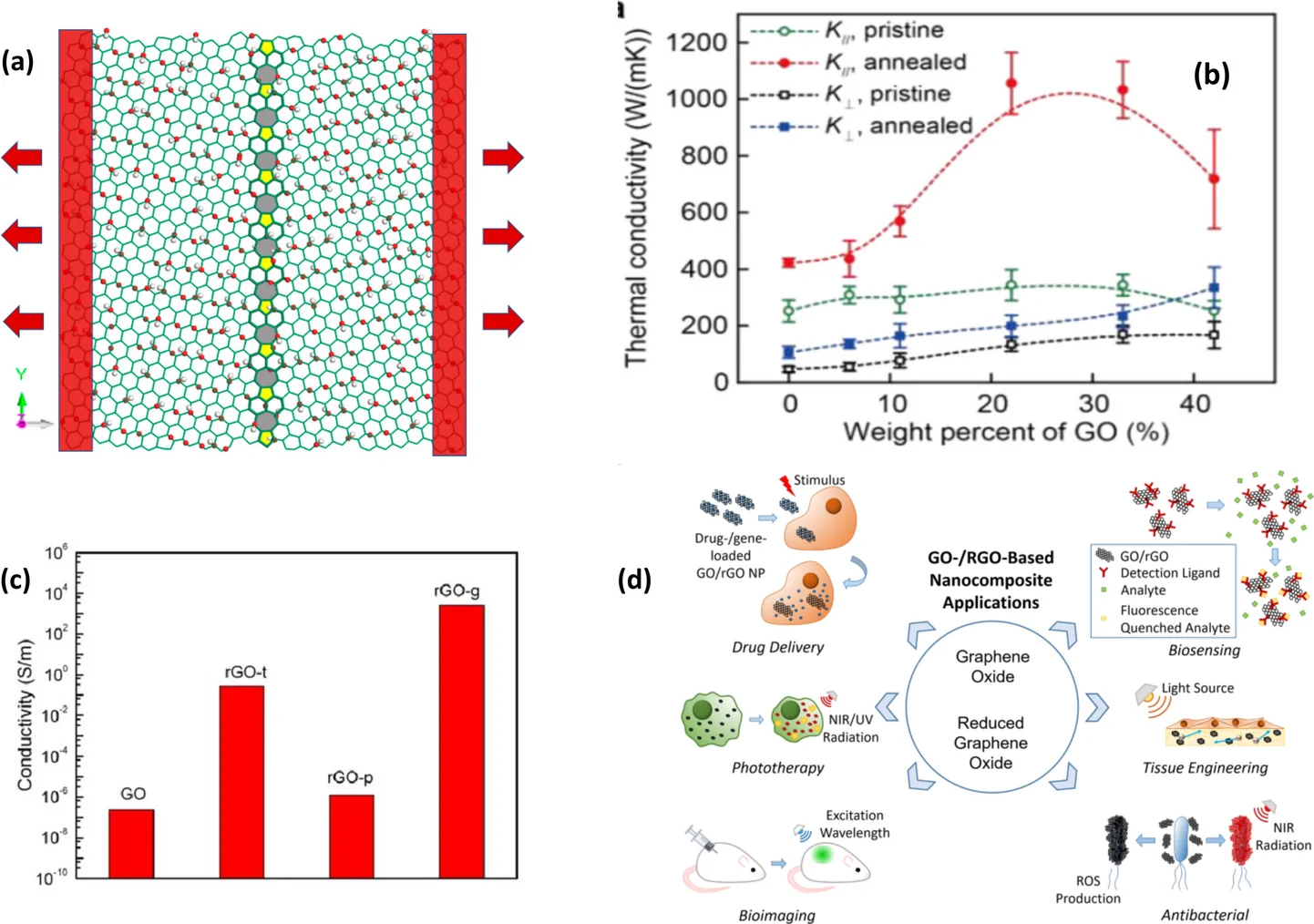
Properties of Graphene oxide **(a)** Mechanical. Produced under common creative license 4.0 [73]. **(b)** Thermal [74] **(c)** Electric conductivity. Reproduced with permission from [75]**(d)** Biological properties. Produced under common creative license [76]

2D planar sheets of sp^2^ hybridized carbon atoms organized in a hexagonal lattice, marked by very strong covalent bonds, make up perfect graphene as shown in Fig. 3 (a). The remarkable slippery elastic behavior and nonlinear properties of graphene are well-documented. When the inherent strength reaches a critical stress level of around 130 GPa, damage starts to occur [71]. The structural features of graphene give it remarkable mechanical capabilities, which contribute to its reputation as an extraordinarily strong material. One further thing that makes graphene perfect for electronics and high-performance sensors is its ability to be flexible. According to a study, the elastic modulus of GO paper is 32 GPa, and the break strengths are around 80 MPa [72].

The small amount of graphene in the polymer matrix gives GBC its remarkable mechanical properties, including increased tensile strength and elastic modulus. Graphene derivative (graphene’s inherent properties), loading value, polymer type, and technique all play important roles in the enhancement [77–79]. Several tests have shown that adding graphene filler to the GBC significantly improves its mechanical properties. The mechanical properties of graphene–polymer composites typically enhance with higher graphene concentration until an optimal concentration is reached, after which filler aggregation results in performance decline [80]. Bao et al. examined two composites formulated with a PVA matrix incorporating 0.8 wt% graphene and 0.8 wt% reduced graphene oxide (rGO), respectively, and reported that the rGO/PVA composite demonstrated much superior mechanical abilities [81]. The findings demonstrate that rGO/PVA significantly enhanced mechanical characteristics when compared to GO/PVA. In comparison to GO/PVA, which showed gains of 54% in tensile strength and 52% in elastic modulus, rGO/PVA showed an increase of 66% in both metrics. Theoretically, this was due to the greater mechanical strength of rGO. Graphene oxide is an excellent addition for polymers because to its functional groups, which enhance mechanical characteristics, facilitate load transfer and interfacial interactions, and display good dispersion inside the polymer [78]. Furthermore, the method affects the ultimate composite characteristics in the opposite way. Specifically, 1 weight% rGO/PMMA composites produced by in situ polymerization and solution mixing demonstrated a 60.7% and 20% improvement in tensile strength, respectively [82, 83]. The GBC has shown promise in a variety of materials worldwide that require high mechanical strength, low weight, and high flexibility [78, 84].

### Thermal Properties

Since non-doped graphene has a low density, phonon transport governs thermal conductivity rather than electronic conductivity, as shown in Fig. 3 (b) [85]. Theoretical and experimental investigations confirmed that graphene has 3000–5300 W/m K thermal conductivity. While comparable to carbon nanotubes, this value was much higher than that of diamonds and graphite. Effective heat conduction may be shown by gate vibration or phononic models through the sp^2^ hybridization of strong C-C bonds [86].

Theoretically, a composite with better thermal properties can be made by mixing a small amount of graphene with polymers [78, 84]. Numerous studies have examined the thermal stability and conductivity of graphene oxide-based materials, including 25% rGO-Epoxy, 0.3% rGO-Epoxy by weight, 2% rGO-PS, 0.7% GO-PVA, and 3% rGO-PLA [87–91]. A 25 vol% rGO filler in epoxy may increase the heat conductivity by almost 3000%. According to a study, the composites synthesized by Yu et al. outperformed traditional carbon material fillers in terms of thermal conductivity, with a value of k = 6.44 W/m K. The composite’s heat conductivity may be limited, nevertheless, due to the filler-matrix contact. Graphene oxide was functionalized by Hu et al. to enhance the composite’s interfacial adhesion and solve this problem [92]. Adding 0.5 vol% graphene oxide to the epoxy increased its thermal conductivity by 800%. The composite outperformed carbon nanotube (CNT) infused composites in terms of heat conductivity by 30%. In contrast to CNTs, which have a one-dimensional structure, Bui et al. showed that graphene oxide-based materials (GBC) exhibit higher thermal conductivity than graphene’s two-dimensional structure [93].

Adding graphene or a derivative to a polymer matrix composite may increase the composite’s thermal stability and flame retardancy. This takes into account graphene’s exceptional thermal stability as a barrier that prevents combustion gas transfer. Compared with a polymer matrix without graphene, GBC with a small amount of graphene added might exhibit much better thermal stability [79]. Guo et al. combined epoxy with reduced graphene oxide (rGO) to form a composite, which was investigated for its combustibility [88]. At 5 wt% rGO, the results showed a 43.9% drop in the total heat release rate. According to a study, GBC can be used in heat applications, including as electronic components, due to its conductivity and stability [79].

### Impermeability

Because of its (i) high electron density, (ii) densely packed carbon atoms, (iii) firmly overlapped sheets, and (iv) high resistance, graphene is impenetrable to several gases and compounds. One of the most impermeable materials known to science is graphene. There were claims made in 2008 regarding potential new uses for graphene-based materials’ impermeability, including selective gas permeation, preservation of water molecule layers, confinement in wet electron microscopy, nanoporous bio-scale structures, and ultra-thin composite corrosion coatings. For permeability, the selected synthesis technique is critical as it influences flaws [94]. Adding graphene to PVA significantly increased its water impermeability, as reported by Wang et al. [95]. Composites made with 1wt% rGO had a water absorption ratio of 48.8%, down from 105.2% for pure PVA. Not only that, GBC has excellent barrier capabilities, such as being impermeable to gases like O_2_, N_2_, and He [96–98]. Biological devices and packaging materials rely heavily on gas and water barrier properties [78, 99].

### Electrical Conductivity

More people are interested in graphene’s electrical characteristics than in its other amazing features. Graphene has a distinct configuration of Fermi energy levels and a linear Dirac-like spectrum, suggesting that it may be an inherent semiconductor as shown in Fig. 3 (c) [100]. The parabolic spectrum of double-layer graphene is very tiny and insignificant, with a band overlap of 1.6 meV. So, it may be thought of as a metal with a special form of exciton. An excess of three layers of graphene belt should be shown by many load carriers across the valence and conduction bands [101, 102].

Adding graphene to polymers to enhance their electrical characteristics has been the subject of many studies. 1 vol% rGO-HDPE, 2 wt% rGO-PU, 3–5 wt% rGO-PLA, and 1 vol% rGO-PS were among the tested graphene oxide-based composites (GBC) [103–106]. A GBC with as little as 1 vol% rGO reportedly exhibits electrical conductivity ten times higher than pure polystyrene (PS) [106].

An ideal electrically conductive GBC has a filler network that is both effective and conductive [78]. This ensures improved conductivity with less graphene filler, which is the lower limit for the insulator to conductor transition. The lower limit for the volume of graphene in the GBC affects the economic feasibility for maintaining the processability of the composite and the cost of the components. The electrical properties of graphene/polymer composites are influenced by the aspect ratio, dispersion state, contact resistance, the quality of the connected 3D network, and intrinsic electrical conductivity. Furthermore, GBC shows improved electrical conductivity over CNT-based composites. Due to graphene’s properties, including (i) an increased surface area impediting agglomeration, (ii) a lower aspect ratio from wrinkling and crimping, and (iii) a 2D planar structure resulting in multiple contact points, GBC can be used in a variety of applications that require electrical conductivity [78].

### Biological Characteristics

The majority of graphene oxide and its derivatives have demonstrated cytocompatibility both in vitro and in vivo, as shown in Fig. 3 (d). On the other hand, 2D materials may affect cellular function due to their physicochemical characteristics. There are two aspects to the functioning of graphene surfaces: surface concentration and aggregation states. When transported across cell membranes, graphene’s rough edges might damage cells. Its aggregated form has the potential to cause cell death in some cases. Due to decreased steric hindrance, nanoscale graphene is related to cytotoxicity, inflammation, and genotoxicity, all of which are undesirable results. In addition, cells quickly absorb functionalized graphene (GO, FGO, and rGO), particularly at nanoscale levels, which causes greater breakdown of cell membranes. There are tissue engineering uses for graphene oxide and its derivatives, which also have antibacterial characteristics. A great deal of research has focused on the processes that allow graphene oxide to exert its antibacterial properties, such as photo-thermal effects, basal plane, oxidative stress, entrapment, and membrane tension [107]. The sharp edges of GO have the potential to damage cell membranes, which might trigger the membrane stress response system. For some bacteria, such as *Pseudomonas aeruginosa* and *Staphylococcus aureus*, the GO structure enables electron abstraction at the cell membrane, weakening the cell wall and ultimately killing the organism. In particular, *E. coli* and other bacteria are susceptible to oxidative stress and ultimate death when exposed to GO and rGO because of the functionalized groups on their surfaces, which may change the intracellular partial pressure of oxygen. Photothermal therapy uses graphene oxide to enhance antibacterial activity, as the two substances exhibit synergistic effects when combined with laser light. An important reason why GO-based materials have antioxidant action is that they need sp^2^ carbons for electron transfer and radical adduct formation [108, 109]. Because of this quality, these biomaterials have the potential to protect cells from oxidative stress by effectively neutralizing free radicals. Toxic effects, immune system reactions, and environmental risks are major concerns with non-biodegradable graphene (not including FGO). In an oxygen-rich environment, GO can be oxidized by horseradish peroxidase and hydrogen peroxide. Biomaterials, like scaffolds, need to degrade at the same rate as tissues and organs; hence, there have been several attempts to speed up the biodegradation of GBCs [110]. Functional groups on the surface of GO significantly increase the bioactivity of polymers, as shown by Wan and Chen [111]. This led researchers to conclude that GO’s beneficial bioactivity was responsible for the calcium phosphate buildup on the GBC surface. Graphene oxide-based materials have been developed as viable alternatives for biomedical applications owing to their superior biocompatibility and the ability to be functionalized with biomolecules, thereby enhancing their specificity and efficacy. Their effective incorporation into drug delivery systems facilitates the regulated and prompt release of therapeutic agents, tackling issues associated with medication stability, bioavailability, and targeted delivery [112].

### Biocompatibility and Toxicity

Biocompatibility remains a critical determinant for the clinical translation of graphene oxide (GO)-based nanomaterials. While GO offers exceptional surface chemistry and dispersibility, its toxicity profile is highly context-dependent, influenced by physicochemical parameters (lateral size, oxidation degree, surface functionalization), exposure route, and biological model. Recent systematic evaluations emphasize the need to distinguish in vitro cytotoxicity from in vivo outcomes and to address dose-dependency and regulatory hurdles [113].

In vitro findings consistently demonstrate dose-dependent cytotoxicity, primarily driven by oxidative stress, membrane disruption, and ROS generation. Pristine GO typically induces significant cell viability reduction above 50 µg mL⁻¹ in epithelial, macrophage, and cancer cell lines, whereas functionalized GO (PEGylated, protein-coated, or reduced forms) shows markedly improved cytocompatibility, with viability often exceeding 90% even at higher concentrations [114]. In vivo studies, however, reveal a more nuanced picture. In rodent models, high-dose GO administration can trigger acute pulmonary inflammation, fibrosis, and organ accumulation in a clear dose- and time-dependent manner. In contrast, recent large-animal data are far more reassuring: a 2025 pilot study in swine demonstrated that water-dispersible pristine graphene and GO elicited only modest local tissue reactions following intraperitoneal injection, with no evidence of systemic toxicity or significant immunological activation [113].

Most notably, the first-in-human controlled inhalation trial exposed healthy volunteers to thin, highly purified GO nanosheets for two hours under double-blind conditions; no adverse effects on pulmonary function, cardiovascular parameters, or systemic inflammation markers were observed, establishing acute tolerability in humans for the first time [115]. Dose-dependency emerges as the dominant factor across both in vitro and in vivo settings: low-to-moderate doses (< 10–50 µg mL⁻¹ or equivalent in vivo) are generally well-tolerated or even bioactive (e.g., promoting cell adhesion), while higher exposures consistently exacerbate ROS-mediated damage, genotoxicity, and chronic inflammation. Surface modification and controlled lateral size (< 1 μm) further reduce these risks by enhancing biodegradability via neutrophil myeloperoxidase and enzymatic pathways [113]. Regulatory concerns centre on the absence of standardized testing protocols, batch-to-batch variability, long-term biodistribution/clearance data, and scalability under Good Manufacturing Practice (GMP). Agencies such as the FDA and EMA require harmonized nano-specific guidelines for nanomedicines, including chronic toxicity, immunogenic potential, and environmental impact assessments—areas where GO currently lacks comprehensive datasets [115].

## Healthcare Sectors and Biomedical Applications

### Biocompatibility of Graphene Oxide-Based Materials

Graphene oxide has emerged as a very adaptable nanomaterial for biomedical applications due to its extensive specific surface area, many oxygen-containing functional groups, superior dispersibility in aqueous environments, and straightforward surface functionalization [116]. It is essential to evaluate the biocompatibility, toxicological effects, and potential environmental impacts of graphene and its byproducts before integrating it into human tissue, particularly for implant applications. Much excitement surrounds graphene oxide-based sensors and their potential uses in biotechnology and health monitoring. There have been a lot of studies done on graphene oxide-based nanomaterials [117]. To this day, there has been no comprehensive analysis of the effects on ecosystems and human health. For new materials, thorough safety evaluations are necessary. Biosensors are analytical tools that combine biophysical detectors with functional chemicals or biological components to detect biological analytes. This device can convert your biological data into a more measurable signal. The durability, environmental sensitivity, and remarkable biological compatibility of graphene oxide make it an ideal material for real-time sensing devices and point-of-care applications [118]. Chemical sensing is one of the fastest-growing industries, and new materials are crucial for its success. Graphene oxide meets all the requirements to produce efficient sensors because of its extraordinary characteristics. It is quite challenging to implement new sensory systems that can replace its unique properties. The electrical and optical properties of a single living molecule or biomolecule on or near the graphene oxide surface may be altered by charge or energy transfer produced by localized deformation [119]. Graphene Oxide contains several oxygen-containing functional groups (including hydroxyl, epoxide, and carbonyl) predominantly at the edges and surfaces, significantly influencing sensor performance via chemical interactions, adsorption, and charge transfer. The ripples, residual functional groups, layers, and sheet dimensions significantly affect the sensitivity and selectivity of GO-based sensors [120, 121]. Research indicates that surface properties, including atomic-level bonding configuration and nanoscale morphology, markedly affect the biological activity of graphene oxide-based materials by improving hemocompatibility, cellular adhesion, and proliferation, underscoring the significance of surface engineering in the development of clinically relevant biomaterials [122].

Graphene oxide (GO)-based nanomaterials remain largely preclinical, with limited progression to human studies. The landmark first-in-human trial involved 14 healthy volunteers who inhaled thin, ultra-pure GO nanosheets (200 µg m⁻³) for two hours under double-blind, controlled conditions. No adverse effects were observed on lung function, blood pressure, heart rate, or systemic inflammation markers, demonstrating acute tolerability of carefully designed GO (ClinicalTrials.gov: NCT03659864) [123].

In the field of neurosurgery, interim findings of the first-in-human clinical trial (NCT06368310) using graphene-based cortical electrodes for brain tumor resection surgery, conducted by INBRAIN Neuroelectronics, have been published. Neuroelectronic (2025) published findings from the first four patients that resolved the hypothesis about device-associated negative events. Additionally, the findings demonstrated excellent signal resolution, including the resolution of high-frequency gamma activity, and the intraoperative performance of the electrodes was excellent and safe [124]. In spite of excellent safety findings, there have been no Phase I/II clinical trials of any GO-based therapeutic products (i.e. drugs, imaging, or scaffolds). Significant barriers still exist in the arena of regulatory affairs. Both the FDA and EMA have no specific approval pathway for graphene. Currently, they review the materials in situ and evaluate extended restrictions by considering general nanomaterial guidelines. These include long-term studies of dissemination in the human body, immunological response assessments, and compliance with conditions of Good Manufacturing Practice (GMP) [125, 126].

### Biological Sensing

Graphene oxide is extensively utilized in biological sensing applications owing to its remarkable characteristics, such as a high surface area, biocompatibility, and the presence of oxygen-containing functional groups that promote strong interactions with biomolecules [127]. GO effectively extinguishes fluorescence through energy transfer, facilitating the sensitive detection of DNA, RNA, proteins, and tiny molecules by observing fluorescence variations. It also serves as a matrix to enhance the ionization of biomolecules for sensitive mass spectrometry-based identification of diverse bioanalytes [128]. The hydrophilicity and biocompatibility of GO facilitate robust, precise interactions with nucleic acids, proteins, and other biomolecules. The adjustable surface chemistry enables the development of hybrid nanocomposites designed for various biosensing platforms. These attributes of GO provide ultrasensitive, real-time detection appropriate for medical diagnostics, environmental surveillance, and food safety [129]. Hasler et al. developed clickable graphene nanoribbons for use in electrochemical sensors that detect interleukin 6 by using nanoribbons as interface linkers for rGO-based field-effect transistor sensors [130].

### Graphene Oxide-Based Materials for Sensing Biomolecules

#### DNA Sensing

This innovative detection device, which uses water-soluble graphene oxide as a structure for precise DNA identification, is simplified in the following diagram. GO can fully obstruct the fluorescence of dye-labeled sDNA by binding to it, given that GO has been demonstrated to exhibit strong noncovalent interactions with nucleobases and aromatic compounds. The binding of dye-labeled DNA changes the shape of the target molecule, which disturbs the interaction between GO and the dye-labeled DNA when a target is present. When the dye-labeled DNA is released from the GO via interactions, its fluorescence is restored. Because of its sensitivity and specificity towards the targeted molecule, this approach has the potential to deliver fluorescence-enhanced sensing. Graphene oxide found in this research is a good platform for sensitive, targeted biomolecule detection, just like carbon nanotubes. However, GO is a better material for biosensor development than CNTs because it is cheaper and can be produced at an enormous scale [131, 132]. Liu et al. designed a fluorescent aptamer sensor utilizing graphene oxide (GO) for the detection of thrombin. Fluorescein-labeled thrombin aptamer was formed in Tris-HCl buffer and combined with thrombin for 5 min at ambient temperature. The hybridization process lasted approximately 30 min, resulting in fluorescence recovery due to the aptamer-thrombin interaction. The fluorescence was observable within a spectrum of 1 nm to 1 mm. The sensor provided a sensitive, specific FRET-based approach for thrombin detection, exhibiting significant signal changes upon interaction [133].

#### Protein Sensing

Graphene oxide (GO) is considered a significant material for protein sensing because of its extensive surface area, superior water dispersibility, and extensive oxygen-containing functional groups that promote conjugation with biomolecules [134]. Its excellent fluorescence quenching potential and facilitation of electron transfer make it optimal for developing sensitive and selective biosensing systems [135]. GO interacts with target proteins primarily through hydrogen bonding, electrostatic interactions, and π–π stacking, facilitating detection via several techniques, including electrochemical methods, fluorescence resonance energy transfer (FRET), and Raman spectroscopy [136, 137].

**Fig. 4:**
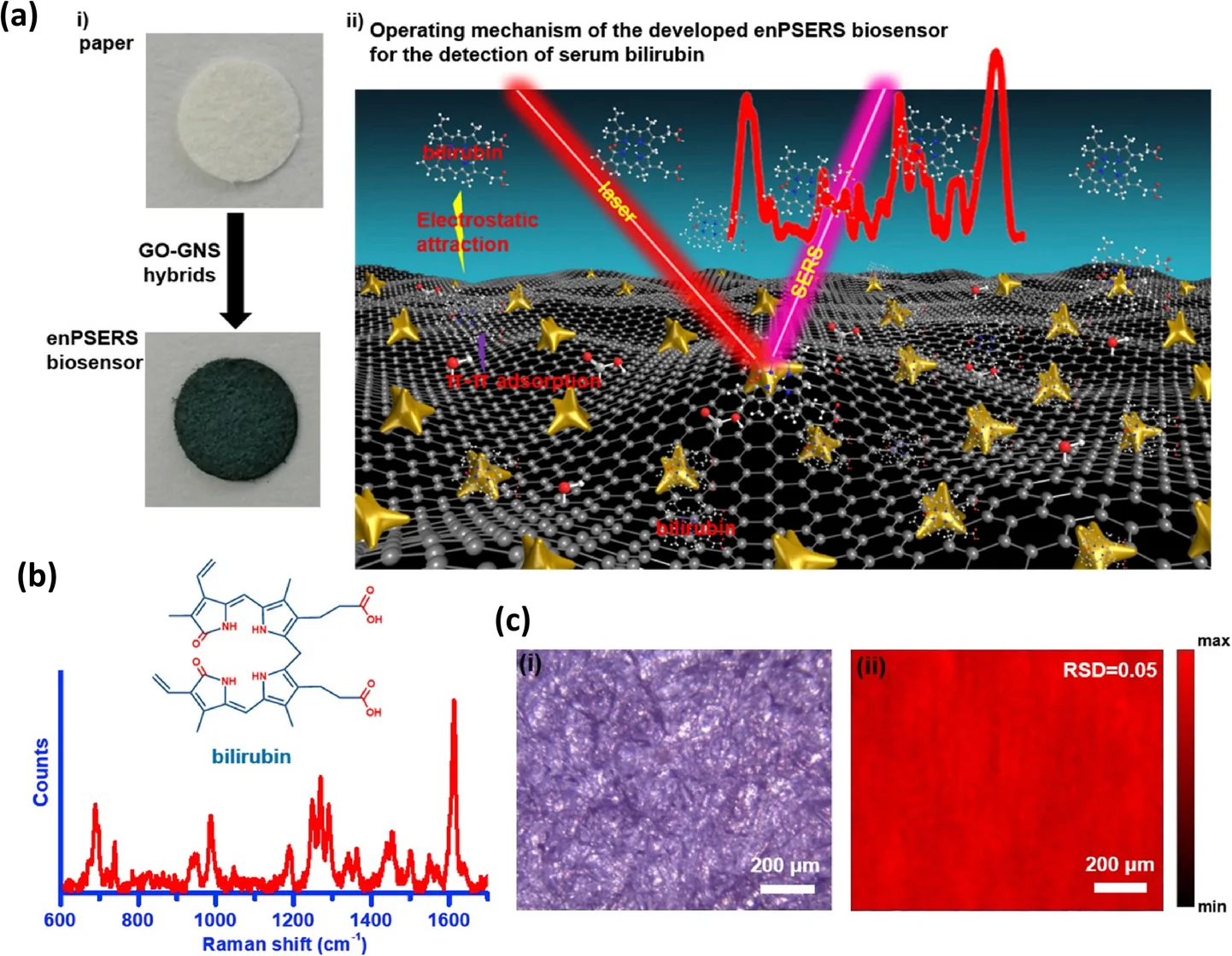
Design and implementation of the enPSERS biosensor for label-free determination of bilirubin in serum. **(a) **(i) A schematic showing how the enPSERS biosensor is made and how it works for label-free SERS detection; (ii) the working principle of the biosensor. The enPSERS biosensor was made by combining the filter paper with the GO-GNS hybrids. **(b)** The molecular structure and Raman spectra (solid) of bilirubin are visible in both the optical image and the corresponding SERS image of the enPSERS biosensor. **(c) **(i) Optical and (ii) SERS image of the enPSERS biosensor with 4-NTP at a concentration of 1.0 µM. The mapping of the N–O Raman band at 1328 cm⁻¹ throughout a 1200 μm × 1000 μm area reveals consistent signals (RSD = 0.05), hence validating homogeneous GNS decoration and superior sensor quality. Reproduced with permission from [138]

A new photoluminescence test for measuring protein kinase activity has been developed by researchers. It uses graphene quantum dots and is both fast and accurate. Using a new approach that depends on the targeted buildup of phosphorylated peptide-GQDs caused by the coordination of Zr^4+^ ions, a kinase-catalyzed phosphorylation of peptides was observed. In contrast to the conventional FRET quenching procedures employed in enzyme tests that rely on fluorescence, this approach offers a novel and attractive alternative. Prompt detection (within 5 min) and an easy investigation procedure that avoids complex protein identification processes are only two of the many benefits of this technology, which uses peptide-conjugated GQDs, over existing fluorescent kinase approaches. Development of kinase-related drugs and assessment of kinase inhibitors are two areas that might benefit from the suggested approach. Pan et al. designed a paper-based SERS biosensor (enPSERS) utilizing graphene oxide–gold nanostar (GO-GNS) hybrids for the sensitive, label-free detection of free bilirubin in blood serum. The GO-GNS exhibited elevated SERS sensitivity, bilirubin enrichment, and fluorescence quenching, with adsorption governed by electrostatic and π–π interactions, as described by a pseudo-second-order kinetic model. The biosensor attained two linear detection ranges (5.0–150 µM and 150–500 µM) with a minimal detection limit of 0.436 µM and showed high concordance with the commercial diazo technique, indicating its potential for precise clinical diagnosis of jaundice. Figure 4 (a) illustrates the fabrication of the enPSERS biosensor, wherein GO–GNS hybrids (121% GNS: GO) were applied on filter paper, resulting in a dark blue hue attributed to the hybrid coating. Figure 4 (b) shows the SERS mapping results utilizing 4-NTP, exhibiting consistent Raman signals across an extensive area with minimal fluctuation (RSD ~ 5%), and Fig. 4 (c) presents the optical image and the associated SERS image of the enPSERS biosensor utilizing 4-NTP as a probe molecule. The SERS mapping of the N–O band at 1328 cm^− 1^ over a 1200 μm × 1000 μm area demonstrated a consistent signal distribution. The minimal relative standard deviation (RSD = 0.05) validates the uniform distribution of gold nanostars on graphene oxide nanosheets, guaranteeing consistent hot spot formation and illustrating the high quality and reproducibility of the biosensor [138]. Graphene and gold were used to build a biosensor that relies on surface plasmon resonance [139]. A single- or triple-step grating design was chosen due to the fact that the refractive indices of water, glucose, and blood are all different, so that it could detect them more accurately.

#### Bio-Electrochemical Sensors

The big pores improve binding partner detection by allowing the analyte to move more freely. Direct detection systems based on graphene have the potential to discover important biological components even at low concentrations. New electrochemical sensors are showing interest in graphene oxide due to its unique morphological and electrochemical properties. Due to the rapid electron transit rate at graphene’s edges and defects, rGO layers or small pieces of pure graphene are well-suited for use in electrochemical experiments [140]. The inherent catalytic activity of reduced graphene oxide makes it useful for sensor-based enzymatic applications; it produces many minor enzymatic products, including hydrogen peroxide and NADH. Electrodes that are grounded in graphene have a huge operational reaction area and can pack enzymatically because of their extraordinarily high surface-area-to-volume ratio (theoretically 2600 m^2^g^− 1^). The excellent strength-to-weight ratio and potential for percolating charge conduction channels created by even a little quantity of graphene make it a promising candidate for use in functional composites. Reduced graphene oxide is the preferred material for the majority of graphene sensors due to several reasons, such as rGO’s excellent electrochemical performance, which is guaranteed by its abundance of defects and functional groups, surface chemical moieties make it versatile and adaptable for various applications, which improve sensor efficacy, electrical and chemical properties are highly adjustable, and rGO shows effective electrical conductivity, unlike nonconductive GO.

### Bioimaging

Academic and clinical contexts both rely heavily on bioimaging, particularly when trying to understand the inner workings of cells, tissues, and organs, as well as abnormal disease processes. Due to insufficient sensitivity, specificity, and targeting, bioimaging methods have limited medical use. Therefore, it is crucial to develop a range of imaging techniques that are suitable for certain uses. Photography, fluorescence MRI, photoacoustic imaging, and Raman spectroscopy imaging are the four primary imaging modalities that make use of GBNs **(**Table 1**)**.

**Table 1:** The advantages and disadvantages of GO-based nanomaterials in bioimaging

Graphene-based nanomaterials	Classification	Disadvantages	Advantages	References
GO-modified lanthanide nanoprobes	Fluorescence imaging	limited spatial resolution, poor tissue permeability, and reach only the surface layer.	Low cost, excellent sensitivity, multicolor imaging, and easy operation.	[141]
Graphene Oxide Quantum Dots (GOQDs)	Fluorescence imaging	Synthesis complexity, Potential for photobleaching under prolonged illumination	Excellent water solubility, biocompatibility, and strong fluorescent emission are suitable for long-term cellular imaging.	[142, 143]
GO-Fe_3_O_4_ Nanocomposite	Magnetic Resonance Imaging	Cytotoxic, mitochondrial stress, and increasing reactive oxygen species	High transverse relaxivity, magnetic targeting, and improved stability	[144, 145]
Graphene Oxide-Cobalt Ferrite	Magnetic Resonance Imaging	Induction of oxidative stress, inflammatory effect, and the risk of cobalt ion release	Resistant to oxidation, very high T_2_ relaxivity, and better stability in vivo	[146]
PEGylated Superparamagnetic GO	Magnetic Resonance Imaging	Slower clearance mechanism requires monitoring for long-term toxicity	Excellent relaxivity and stability in biological systems, non-aggregating in serum	[147]
Ag-GO Ns modified with folic acid.	Raman Spectroscopy Imaging	Only appropriate for in vivo superficial detection and in vitro tissue detection, with extended data collection and processing time	high resolution, quick scanning speed, avoids tissue spontaneous fluorescence issue, qualitative and quantitative localization analysis	[148]
GO-Au Nanocomposites	Raman Spectroscopy Imaging	High cost, complex synthesis	Excellent photostability, minimal toxicity, strong biocompatible plasmonic enhancement	[149]
rGO@AuNs	Photoacoustic Imaging	Low spatial resolution	Lower scattering, high temperature sensitivity, and real-time monitoring	[150]
Liquid Gallium Core @ Reduced Graphene Oxide Shell nanoparticles	Photoacoustic Imaging	Complex synthesis	Excellent photoacoustic signal, Efficient photothermal conversion	[151]

#### Fluorescence Imaging

In fluorescence imaging (FRI) applications, graphene oxide-based materials play a vital role due to their surface and edge defects, suitable band gaps, and outstanding light stability, which give them strong fluorescence characteristics. GO-based materials have limited use in vivo FRI because of their low fluorescence quantum yield. The use of fluorescent dyes for the covalent or noncovalent functionalization of GO is essential to overcome this limitation and enhance FRI signals. GO can serve as a carrier for various photosensitizers in FRI studies [152]. It is possible to monitor the distribution and delivery of medications in vivo in real-time via intramolecular charge transfer, which may substantially improve fluorescence performance. Hydrophilic lanthanum nanocrystals were synthesized and exhibit robust visible/NIR-II luminescence. Encapsulating them in GO allowed them to accomplish intracellular tracking, microRNA-21 detection, and tumor targeting NIR-II imaging in vivo. Additionally, they showed a strong response around 1525 nm.

**Fig. 5:**
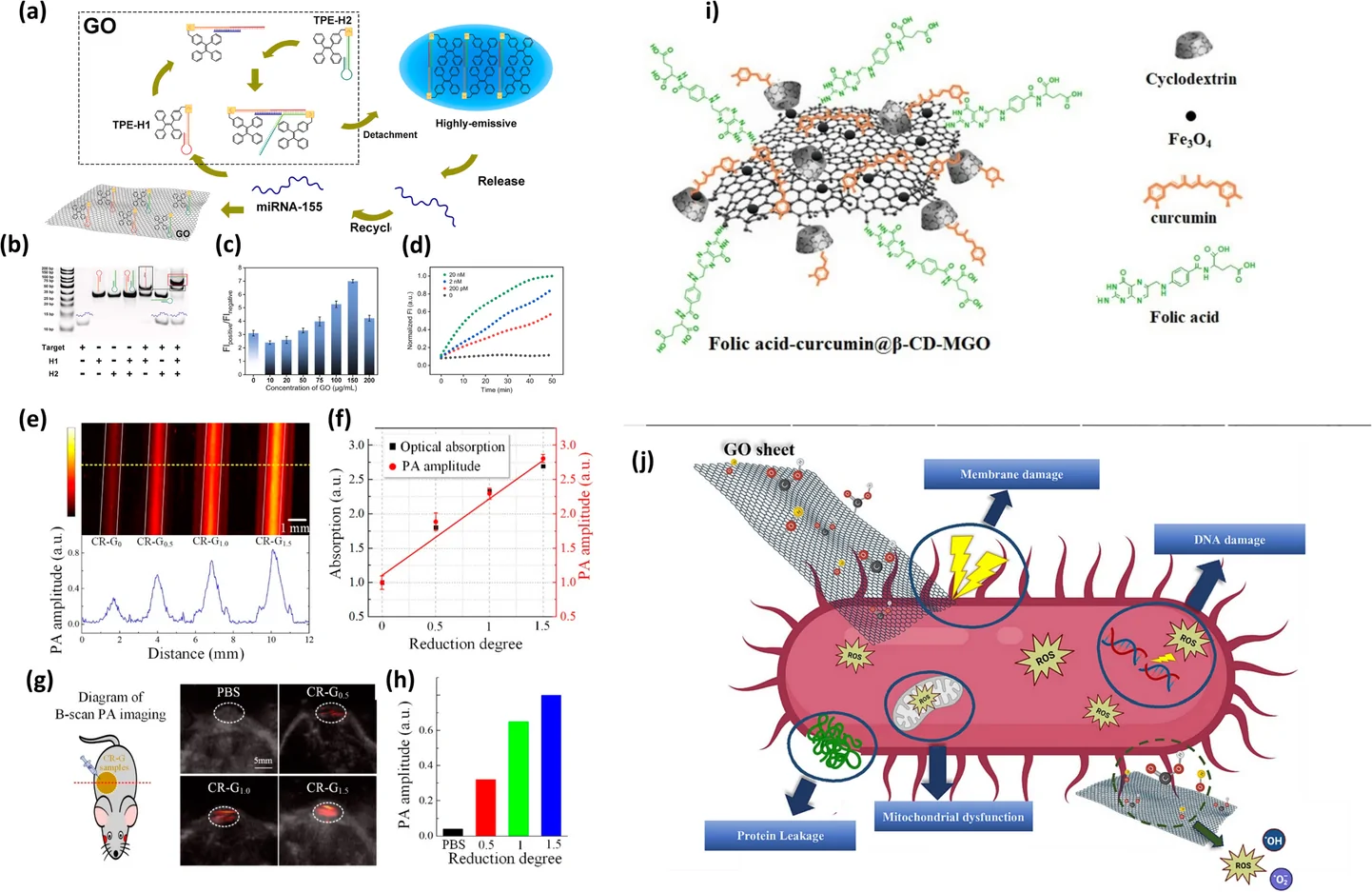
**(a)** A schematic representation of the CHAIE method for detecting miRNA-155 in solution. **(b)** Electrophoresis analysis of the CHA reaction products **(c)** The fluorescence ratio between the target and non-target groups is measured using the CHAIE biosensor with varying concentrations of GO (*n* = 3) **(d)** The kinetics of the CHAIE reaction with the target at different concentrations are presented. Reproduced with permission from [153] **(e)** In vitro PA imaging and signal amplitude profiles of CR-G nanomaterials with varying degrees are shown. A comparison of optical absorption and PA signal amplitudes of CR-G nanomaterials with different reduction degrees in the NIR-II region is provided **(f)** Statistical analysis of the PA signal amplitudes obtained from the different reduction degrees **(g)** B-scan in vivo PA imaging, overlaid with ultrasound imaging, for the mouse model injected with CR-G nanomaterials having different reduction degrees in the NIR-II region **(h)** Statistical analysis of the PA signal amplitudes. Produced under common creative license 4.0 [154]. **(i)** A schematic illustration of the Folic acid-curcumin@β-CD-MGO complex. Reproduced with permission from [155] **(j)** Schematic representation of the antibacterial mechanisms of GO/disopside. Reproduced with permission from [156]

Song et al. reported an intelligently assembled biosensor based on catalytic hairpin assembly (CHA) and aggregation-induced emission (AIE), referred to as catalytic hairpin aggregation-induced emission (CHAIE), for the ultrasensitive detection and intracellular imaging of miRNA-155. Both the detection sensitivity of miRNA liquid biopsy and intracellular imaging are hindered by the low abundance, diverse expression, and temporal variations of miRNAs across distinct cellular sites. These issues were overcome by them. To do this, graphene oxide was used to quench the fluorescence, and tetraphenylethylene-N3 was utilized as the AIE luminogen. Because the CHA reaction was initiated when the target miRNA was present, the AIEgen was able to self-assemble with the hairpin DNA. A robust AIE fluorescence signal was created, and the intramolecular rotation of the AIEgen was limited by this arrangement. The CHAIE biosensor was notable for its quick detection since it did not need enzymes or costly heat cycling equipment. The biosensor detected miRNA at concentrations ranging from 2 pM to 200 nM in as little as 30 min under ideal conditions, with a limit of detection of 0.42 pM, as shown in Fig. 5 (a-d). Due to its great sensitivity and minimal background signal, the CHAIE biosensor is ideal for accurate spatiotemporal imaging of target miRNA in live cells [153].

#### Magnetic Resonance Imaging

Magnetic resonance imaging (MRI) has replaced other methods in bioimaging because of its noninvasiveness, high spatial resolution, and infinite tissue penetration depth. Manganese and gadolinium are the two most popular contrast agents utilized in MRI scans [157, 158]. However, in vivo, nonselective coordination results in substantial nonspecific toxicity. Chelating harmful paramagnetic metal ions is one way that graphene oxide-based materials with different functional groups lessen their toxicity. The oxygen-containing functional groups on graphene oxide, including carboxyl, hydroxyl, epoxy, and phenolic groups, offer active sites that can bind and stabilize paramagnetic ions such as Gd^3+^, Mn^2+^, and Fe_3_O_4_ nanoparticles. This chelation reduces the release of free ions that commonly induce toxicity in biological systems [159].

Graphene-based magnetic nanocarriers exhibit exceptional performance and biocompatibility for MRI applications. GO-based materials integrated with magnetic nanoparticles, such as iron oxide nanoparticles (IONPs), enhance MRI signals by improving relaxivity and circulation stability. GO-IONP hybrids significantly enhance T2 relaxivity compared to nanoparticles alone, thereby facilitating superior cellular and in vivo imaging [160]. GO composites have been investigated for multifunctional theranostic applications that integrate MRI with medication delivery and photothermal therapy for cancer treatment [161]. Rodriguez et al. indicated that graphene oxide/iron oxide (Fe_3_O_4_) nanocomposites acted as multifunctional MRI contrast agents with less toxicity owing to the chelation of GO of iron ions. The nanocomposites demonstrated augmented MRI signal and superior biocompatibility by inhibiting the release of free iron ions [144].

Su et al. reported a novel strategy for constructing graphene oxide nanomaterials with significantly enhanced photothermal (PT) and photoacoustic (PA) performance in the near-infrared (NIR) II biowindow through chemical reduction. Chemically reduced graphene oxide nanomaterials showed considerable improvement in optical absorption over the whole NIR spectrum, from NIR-I to NIR-II, based on their optical spectra. The improvement was attributed to the graphene oxide sheets reestablishing electronic conjugation, leading to the black-body reintroduction effect observed in most graphite-like materials. The recommended procedure was evaluated by synthesizing CR-G samples with varying degrees of reduction. The testing results revealed that the CR-G samples’ PT performance improved significantly because the chemical decrease increased the absorption of the NIR-II biowindow, as shown in Fig. 5 (e-h). In addition, CR-G samples with varied degrees of reduction were scanned utilizing PA both in vitro and in vivo, demonstrating better NIR-II PA capabilities. As a consequence of chemical reduction, graphene oxide nanoparticles have immense potential for PT and PA applications in the near-infrared (NIR-II) biowindow, and this study provides useful guidelines for their rational design [154].

#### Photoacoustic Imaging

Noninvasive photoacoustic imaging uses tissue or contrast agent absorption of electromagnetic waves of low-power, non-ionizing laser light. After that, it uses visual processing to gather sound waves, then uses focused heating to create broadband sound waves by converting light waves into sound. The biomedical community views photoacoustic imaging (PAI) as a highly promising imaging modality due to its ability to image deep tissues and organs with remarkable spatial resolution. More precisely localized blood vessels and melanomas may also be seen. Haemoglobin and melanin are examples of endogenous contrast agents (CA) that have a major impact on the results of imaging. Accordingly, CAs play a pivotal role in achieving optimal imaging performance in PAI [162]. Not long ago, diffusion of GO-based nanomaterials in PAI showed remarkable near-infrared absorption. When it comes to deep organs and tissues, GO-based nanomaterials have better spatial resolution than pure FRI. Possible improvements to contrast and speckle are also in the works. Due to its larger sp^2^ domains, rGO exhibits improved absorption of near-infrared light, making it an ideal photoacoustic contrast agent. For photoacoustic imaging and photothermal treatment of pancreatic cancer, the study synthesized a reduced graphene oxide@gold nanostar. Following surface cross-linking on the rGO@AuNS, folic acid binds specifically to tumor cell folate receptors. The imaging performance of receptor-mediated endocytosis and targeting effectiveness are both greatly enhanced by this. The ability of rGO to absorb near-infrared light is connected to the synthetic system’s effectiveness. Regarding the photoacoustic signal, rGO@AuNS outperforms both bare gold nanostars and unreduced graphene oxide. On the other hand, rGO is less hydrophilic than GO, which might lead to worse biocompatibility. A one-step reduction of bovine serum albumin is one possible step in the production of rGO. This leads to rGO with good monodispersion, small size (approximately 70 nm), little cytotoxicity, and exceptional stability. The peak of the rGO PAI signal in cancer in in vivo experiments was at 30 min, and it stayed there for at least four hours [163].

Additionally, the initial graphene might serve as a PAI agent by modifying polymers like PVA or PEG [163, 164]. Graphene can become an ultrasensitive contrast agent due to its notable absorption in the near-infrared (NIR) region when it forms a complex with indocyanine green via π-π stacking interactions [165, 166].

Einafshar et al. presented a novel approach to treating prostate cancer using a nanocarrier composed of folic acid (FA), β-cyclodextrin (β-CD), and magnetic graphene oxide (MGO) as a theranostic agent, as shown in Fig. 5 (i). Curcumin is a potential therapy for prostate cancer, and the carrier was designed to improve its bioavailability and solubility. The overexpression of folic acid receptors on solid tumor surfaces, such as prostate cancer, implies that these receptors might be exploited to direct medicine delivery to tumor cells, lowering the risk of nonspecific adverse effects on healthy tissues. The Folic acid-curcumin@β-CD-MGO nanocarrier was tested in vitro against LNCaP (androgen-dependent) and PC3 (androgen-independent) prostate cancer cells to assess its anticancer efficiency. Cell growth was suppressed, oxidative damage was generated, and apoptotic effects were boosted, with the effects being concentration dependent. Nanoparticle relaxometry indicated that the material might be employed as an MRI negative contrast agent [155]. Overall, this work reveals a promising theranostic agent that increases curcumin distribution, allows for traceability, and promotes its therapeutic potential in the treatment of prostate cancer.

#### Raman Spectroscopy Imaging

Raman spectroscopy relies on molecular vibrational excitation and inelastic scattering of phonons from the sample. RSI is a potent analytical technique that enhances the capabilities of vibrational spectroscopy. It has a good signal-to-noise ratio, little photobleaching, and the ability to multiplex without invasive or harmful procedures. The field of biomedicine makes frequent use of it, particularly in bioimaging. The inherent Raman emissions of GO-based nanomaterials are unique, and it is possible to increase imaging sensitivity by improving Surface-Enhanced Raman Spectroscopy (SERS) by coating GO-based nanomaterials with metal nanoparticles like gold or silver. This property has attracted considerable attention from researchers seeking to use GO as imaging probes in biological cells. Graphene oxide’s in situ reduction of silver ions improved the surface-enhanced Raman scattering of the silver/GO hybrid, which led to brighter and more discernible Raman imaging signals than the original GO. This finding is very noteworthy. The use of folic acid in conjunction with Ag/GO nanoparticles allowed for targeted SERS imaging of cancer cells by targeting the folate receptor [167].

GBNs find use in radionuclide imaging, multimodal imaging, and computed tomography imagery.

Zarei et al. explored the surface functionalization of magnesium (Mg)-based alloys with diopside-graphene oxide coatings to improve the biocompatibility and corrosion resistance of these biodegradable materials for bone tissue engineering. Coatings of varying concentrations of graphene oxide (x = 0, 0.03, 0.06, and 0.12 g/L) were applied to AZ31 alloy via electrophoretic deposition. Surface features, corrosion resistance, bioactivity, antibacterial capabilities, and cytocompatibility of the coated and untreated substrates were the primary outcomes of this investigation. Powdered diopside was produced by means of the sol-gel technique. Among the coatings tested, the Dio/0.06GO nanocomposite exhibited the most compact morphology, as observed by Field Emission Scanning Electron Microscopy. The surface roughness of the Dio/0.12GO sample increased as the GO content increased, according to Atomic Force Microscopy experiments, which also revealed a Sa value of 293.80 ± 23.75 nm as shown in Fig. 5 (j). The wettability of the coatings followed a similar pattern. With a corrosion current density (icorr) 91.5% lower than that of the uncoated Mg alloy, the Dio/0.06GO coating showed the best corrosion resistance. On top of that, after 7 days of submersion in SBF, this coating had the highest Ca/P atomic ratio (1.61), indicating it was the most bioactive of the bunch. The antibacterial activity test revealed that GO had a bactericidal impact on *E. coli* and *S. aureus*, especially at higher doses. Although the Dio/0.12GO coating showed the best MCT3T-E1 osteoblast cellular attachment in vitro cellular assays, the Dio/0.06GO coatings had the greatest cell viability at 92.17 ± 1.2%. It can be concluded that Dio/GO nanocomposite coatings have the ability to enhance the biological characteristics and corrosion resistance of Mg alloy orthopedic applications [156].

### Graphene Oxide-Based Materials for Tissue Engineering and Delivery

Graphene oxide materials have widespread usage in biomaterials applications because of their two-dimensional layered topologies. Multiple studies have shown that GO-based materials have been used in the therapy of numerous medical conditions, as recorded in publications. In vivo bone formation might be enhanced by covering titanium with graphene oxide (GO), which affects the administration of BMP-2 medicine **(**Table 2**)** [168].

**Table 2:** Graphene oxide composite and different biomedical applications

Composite	Drug type	Target cell	Functional	Delivery stimulus	Study	Synthesis	Nano carrier efficiency	Application	Reference
rGO@MSN	Doxorubicin	A549	Drug delivery	pH 5.6, 7.4	In vitro	Hydrothermal	90%	Cancer therapy	[169]
rGO/g-C_3_N_4_/TiO_2_		MC3T3-E1 PC-12	Nanocoating Scaffold	Blue LED	In Vitro	hydrothermal Chemical co-precipitation	50 ng mL^− 1^	Neuronal Bone tissue	[170]
GO@Ge	PAC	L929 MCF7	Phototherapy	pH 5.5, 7.4	In vitro	facile mixing approach	59.86%	Chemo-photothermal therapy	[171]
rGO/MP-pyrene-PEG	-	*E.coli* UT189 *S.Aures*	Antibacterial	NIR	In Vitro	Precipitation	99%	Water infection Biotechnological	[172]
rGO-AuNPs@PCL	Nanofiber Scaffold	S42 PC-12	Neuronal Tissue	-	In vitro	simple bottom-up solution-	0.0005 wt%	Tissue engineering	[173]
GO-PEG-PEI-TPP@ICG	MG63/DOX	Mitochondria	Bioimaging	NIR	In vitro In vivo	Ultrasonication	100 µL	Phototherapy	[174]
GO/Fe_3_O_4_	Methotrexate	Caov-4 HeLa MCF7	-	pH 5.5–7.4	In vitro	Solvothermal	75%	Cancer chemotherapy	[175]
Dex-rGO	anti-DENV drug	Dengue virus	Fluorescence quenching and recovery	-	In vivo In vitro	Facile chemical	< 80%	Antiviral discovery sensing	[176]
Zn-dopamine-rGO	Doxorubicin	T47-D MCF10A	Antibacterial	pH 5.4, 7.4	In vitro	Hydrothermal	99.7%	Cancer treatment Antibacterial	[177]
rGO/PDA	Ara	HeLa	Drug delivery	NIR	In vivo In vitro	Facile method	75%	Gene therapy	[178]
Amine-rGO@Alg/ECM	Hydrogel Scaffold	HUVCE	Cardiac tissue	Dark	In vitro	In-situ Electroactive	25 µgmL^− 1^	Tissue engineering	[179]
rGO-Au	L-cystein	MKN-45	Fluorescence Sensing	-	In vitro	One-pot hydrothermal	0.51 nM detection limit	Cancer Detection	[180]

#### Drug Delivery

A number of biomedical research projects have looked at the potential of GO for drug delivery in recent years. There has been a lot of research into the biological applications of GO-based materials, particularly in smart medication delivery. Strategies for the non-covalent incorporation of medicinal compounds into materials based on graphene include stacking interactions, hydrophobic interactions, and hydrogen bonding [181]. To transport cancer medications that are insoluble in water, Liu et al. suggested using a combination of GO, PEG, and SN38, which shows that the former is soluble in water and that the latter is effective when dissolved in organic solvents. By using the MTS test, NGO-PEG-SN38 was able to effectively eradicate the human colon cancer cell line HCT-116 in vitro. With a half-growth-inhibition concentration of around 10 µM, the water-soluble medication CPT-11 has reduced toxicity [182].

Nanohybrids made of non-genetically-modified organisms and hematin-terminated dextran were used to eradicate drug-resistant MCF-7/ADR cells [183]. Under physiological conditions, NGO-HDex showed better stability than pure NGO. Furthermore, an efficient combination of NGO-HDex with the anticancer drug DOX, with a loading capacity of 3.4 mg mg^− 1^, is possible [184]. Dinda et al. have shown the potential of DOX and ZnS: Mn-doped nanocrystals attached to GO for cellular labeling and drug administration. The maximal DOX trapping efficacy was 100% when 1000 g mL^− 1^ of GO-PEG/ZnS: Mn composite particles were incubated with a 100 g mL^− 1^ DOX solution [185]. Cell death of around 85% occurs in HeLa cancer cells when exposed to the drug-laden DOX nanocomposite at a concentration of 10 g mL^− 1^, in contrast to cells loaded with free DOX, which show cell death of approximately 68%.

Chen et al. created a medication delivery method where a saturation loading capacity of 65% was achieved at a DOX content of 0.3 mg mL^− 1^ in the mixed solution. Using PEG-fGO to create Fe_3_O_4_ nanoparticles. The hybrid shows an enhanced binding ability, attaining a loading capacity of 0.27 mg mg^− 1^, at a 5-fluorouracil concentration of 0.5 mg mL^− 1^ [186]. The hybrid shows an enhanced binding ability, attaining a loading capacity of 0.27 mg mg^− 1^, at a 5-fluorouracil concentration of 0.5 mg mL^− 1^. The use of graphene-based magnetic and plasmonic nanocomposites demonstrated the facilitation of medication distribution using magnetic field.

In summary, up to 90% drug loading capacity and responsive drug release make functionalized GO/rGO platforms even more effective than conventional drug carriers. A main challenge is the reproducibility of the GO/rGO platforms in different batches. Potential toxicity and slow clinical translation are also important challenges. To improve the translation of this technology into the clinic, the focus should be on scalable Good Manufacturing Practice (GMP) production and multi-centric preclinical studies.

#### Gene Delivery

Gene transfer is a good fit for graphene oxide-based materials because of its great transfection capabilities and high loading efficiency. The process of gene delivery involves transferring foreign nucleic acids into eukaryotic cells. The specific method utilized to do this depends on the kind of cell and the goals of the experiment [187, 188]. The recipient cells may express the donated nucleic acid temporarily without reproducing it, or it might be permanently integrated into their genome and replicate with the host genome. Both outcomes can occur during gene delivery. Several techniques are often used to achieve transfection, including electroporation, lipofection, viral vectors, and ballistic particle delivery. The ability to alter the DNA of host cells has far-reaching implications for studying common biological processes, identifying disease molecular origins, and evaluating gene therapy efficacy. In recent years, transfection has gained significant attention due to its widespread usage in studying disease cellular processes and molecular pathways [189]. Recent advances in the field of life sciences have made it feasible to transfect human cells with a variety of nucleic acids. The short non-coding RNAs (siRNA, shRNA, and miRNA) are among them, along with DNA and RNA. Recombinant antibodies, proteins, and viral vectors have been generated by transfection techniques; they have found use in gene therapy, vaccines, and medicines. One must have a solid grasp of the distinction between transient transfection and continual transfection. Through the process of stable transfection, foreign DNA is integrated into the host nuclear genome, enabling the transgene to be produced forever [190, 191]. One may quantitatively assess the transfection effectiveness using real-time polymerase chain reaction, which evaluates the expression levels of certain foreign nucleic acids in a cell as well as other intracellular nucleic acids that may be affected by exogenous nucleic acids, such as microRNAs [192]. Efficient and viable transfection is a standard measure of transfection performance; improved transfection outcomes are linked to these factors. Transfection success is dependent on a myriad of factors, including DNA amount and quality, cell type, cell health, and transfection method. The transfection efficacy is highly dependent on the cell type and its pre-transfection physiological condition. The cells must be in good condition, able to grow, and free of any contaminants.

**Fig. 6:**
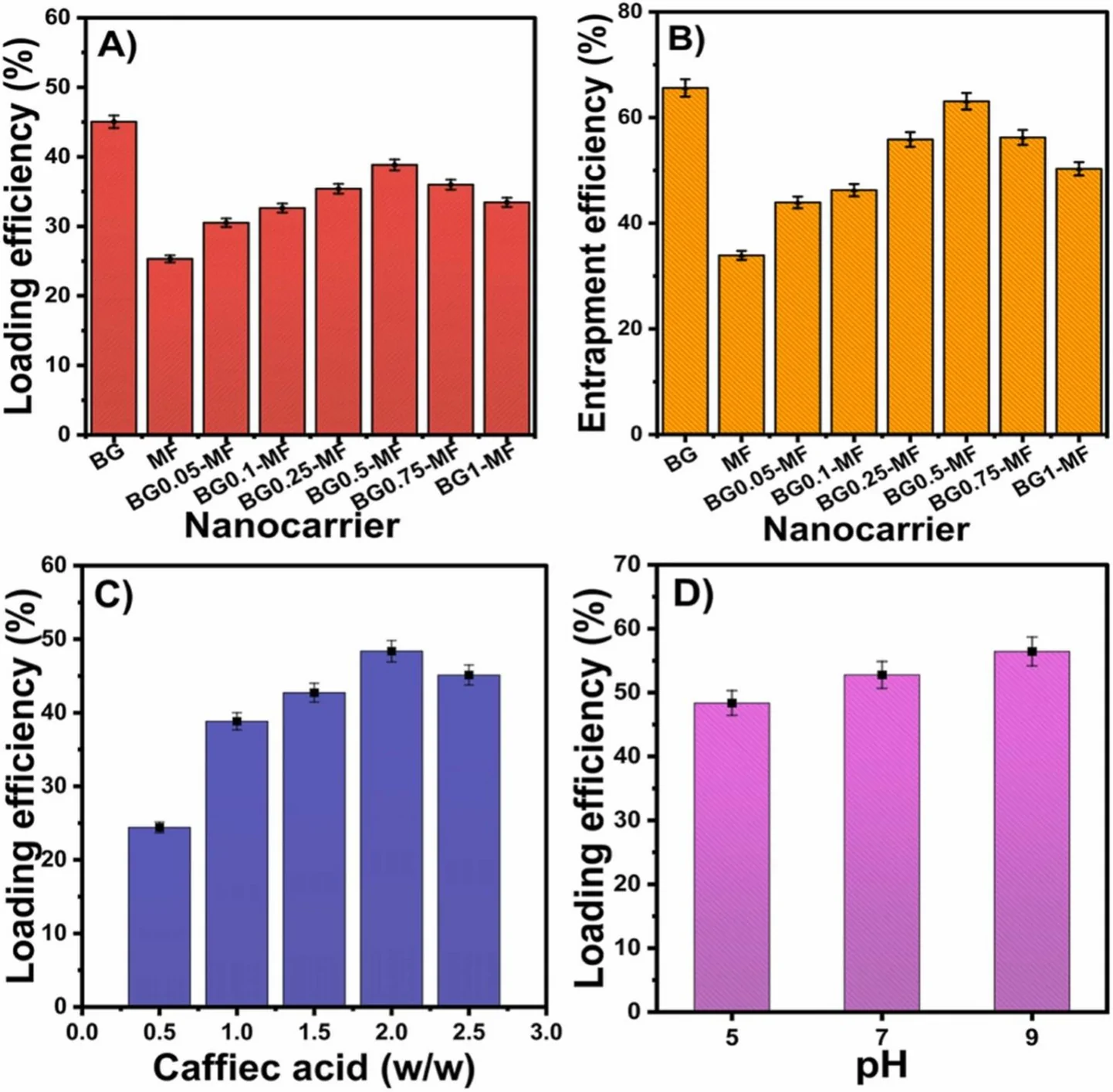
Loading characteristics of CA on βCD-GO nanoconjugate (BG), MF, and BGw-MF nanocarriers. **(a**,** b)** The effect of the amount of βCD-GO in the nanocarrier on CA loading and entrapment efficiencies, respectively **(c)** The effect of the CA: nanocarrier (BG0.5-MF) mass ratio on drug loading efficiency (LE) **(d)** The influence of media pH on the CA loading efficiency (LE) of the BG0.5-MF nanocarrier. Reproduced with permission from [193]

Sontakke et al. presented a novel approach to chemotherapy by utilizing targeted drug delivery systems for improved cancer treatment. Traditional chemotherapy is limited by its inability to selectively target cancer cells, leading to significant side effects. The study focused on overcoming these challenges through the development of self-assembled, sustainable, and straightforward nanocarriers for the delivery of caffeic acid, a natural chemotherapeutic agent. The β-CD-GO@MIL-100(Fe), also denoted as BGw-MF functionalized Fe-based metal-organic frameworks, were used in the synthesis of the nanocarriers, which did not include the use of hydrofluoric acid. The BGw-MF nanocarriers were characterized using a variety of techniques to look at their structural, functional, and morphological properties. The BGw-MF nanocarriers’ CA loading efficiency was measured, which changed with changes in the medium’s pH and the CA-to-nanocarrier ratio. The results demonstrated that BG0.5-MF had the highest loading efficiency, reaching 56.44 ± 2.25% at a 2 w/w ratio of CA in an alkaline environment (pH 9). Figure 6 (a-d) shows that the BG0.5-MF nanocarrier regulated the release pattern of CA, with a release of about 57.07 ± 2.01% at pH 5 and 48.04 ± 1.44% at pH 7.4. This controlled release pattern is advantageous for increasing drug release at tumor locations. Based on the results, BGw-MF nanocarriers might be a great CA-based anticancer DDS. Featuring a regulated release mechanism and a high medication loading capacity, the BG0.5-MF nanocarriers are promising for efficient and sustainable chemotherapy. The cytotoxicity results further highlight the suitability of BG0.5-MF for selective cancer treatment, offering a promising approach for future anticancer DDS [193].

Graphene oxide (GO)-based nanomaterials have emerged as superior non-viral vectors for gene delivery. Their ultrahigh surface area and oxygen functional groups enable high-capacity loading of nucleic acids (DNA, siRNA, mRNA) via π–π stacking and electrostatic interactions, while providing nuclease protection and stimuli-responsive release [194]. Functionalized GO-PEI hybrids demonstrate enhanced transfection efficiency and substantially reduced cytotoxicity compared to high-molecular-weight PEI. Unlike viral vectors plagued by immunogenicity and insertional mutagenesis risks, or liposomal carriers limited by stability and payload capacity, GO platforms offer greater biocompatibility, scalability, and multifunctionality for theranostic applications [195]. Recent studies on PEI-loaded GO quantum dots confirm superior uptake and gene expression in cancer cells with improved safety [194].

Overall, GO-based vectors excel in capacity and performance of both gene loading and gene delivery compared to traditional lipid or viral carriers. The cytotoxicity and inefficient endosomal escape along with the unwanted delivery of genes to other cells, pose challenges. The main effort should focus on biocompatibleGO surface designs validated through in vivo experiments.

#### Tissue Engineering

Repairing injured tissues and organs is the goal of tissue engineering, which entails creating artificial versions of these structures. As biomaterials advanced, they adapted to incorporate scaffolds, cells, and physiologically active substances into living tissues. Graphene oxide and its derivatives are a new class of materials that have the potential to revolutionize tissue engineering and healthcare applications. Shin et al. changed GO-GelMA hydrogels to improve cellular spreading, alignment, survival, and proliferation in a three-dimensional environment [196]. By combining the mechanical and electrical features of this GO reinforcement with a multi-stacking process, an easy way to create complex artificial tissues with cellular functioning and mechanical robustness may be achieved. Tissue engineering and regenerative medicine are both advanced by using GO as a protein-delivery vehicle within a 3D culture system for stem cell differentiation [197].

Making standing hydrogels of graphene oxide and hydroxyapatite is a simple process when it comes to making bone tissue [183]. One potential use of artificial hydrogels is the creation of artificial bone tissue, according to their remarkable characteristics [184]. Coating cracks and delamination were both reduced by using GO sheets in the sintered HA layer. Coatings made of GO and HA are more resistant to corrosion and have better adhesion strength than coatings made of pure HA [185]. Luong et al. assessed hydrogels containing graphene oxide (GO) and poly(ethylene glycol) methyl ether methacrylate (MEO2MA) for applications in wound healing. It was observed that GO-loaded hydrogels increased tensile strength (up to 626 kPa), appropriate elasticity, and significant swelling behavior, all of which are advantageous characteristics for skin tissue scaffolds. Biocompatibility was proven by non-toxic reactions in human dermal fibroblasts. In a rat wound healing model, the GO/MEO2MA hydrogels attained over 95% wound closure within 15 days, markedly surpassing the non-GO controls. Histological study revealed enhanced tissue regeneration, underscoring the therapeutic promise of GO-based wound dressings [198].

**Fig. 7:**
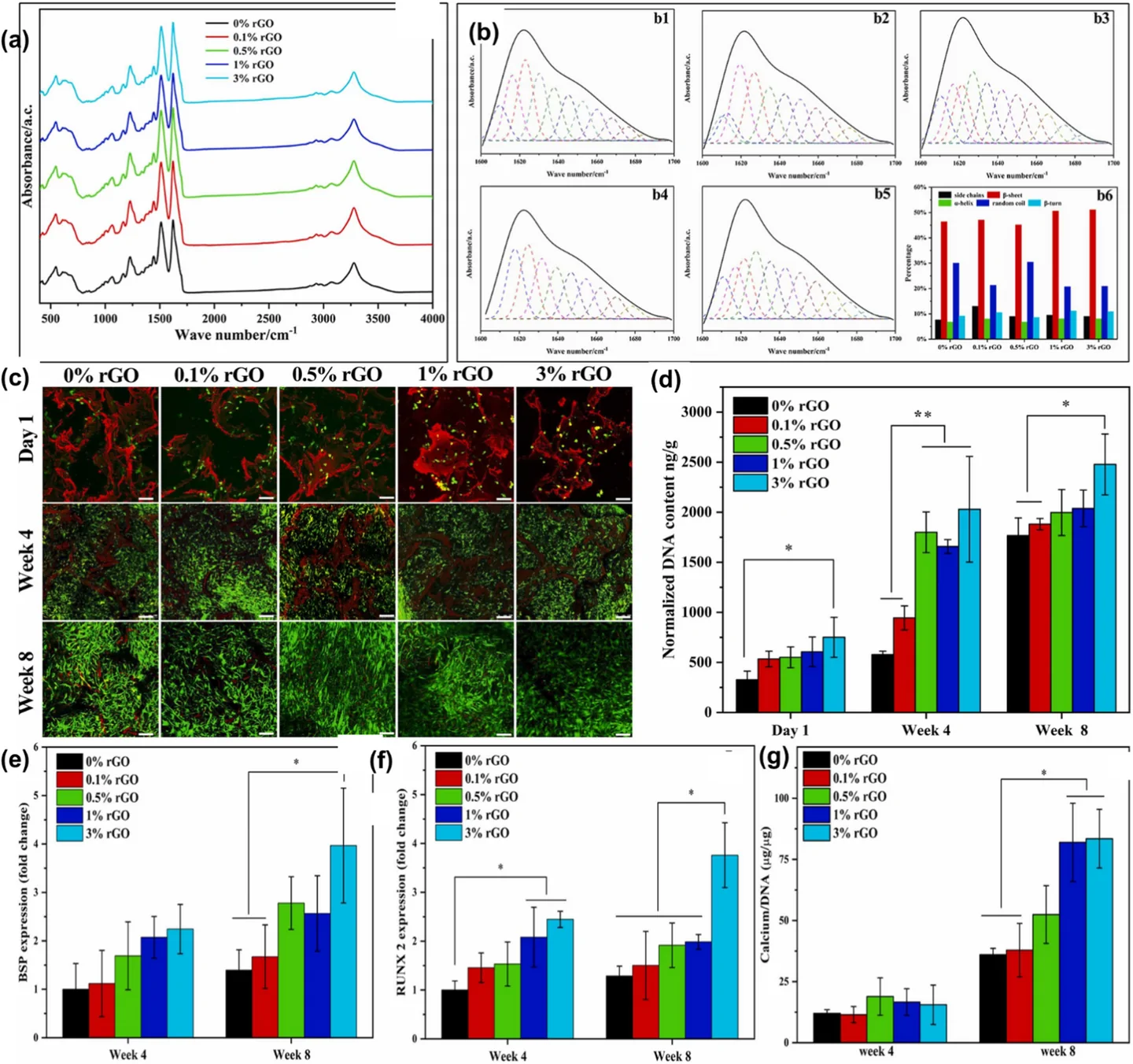
FTIR spectra of the rGO/SF scaffolds **(a)** 4000 cm^− 1^–400 cm^− 1^ **(b)** in the range of amide I region (b1) 0% rGO/SF (b2) 0.1% rGO/SF (b3) 0.5% rGO/SF (b4) 1% rGO/SF (b5) 3% rGO/SF scaffold (b6) amount of secondary structure in rGO/SF scaffolds. In vitro examination of cell proliferation on rGO/SF scaffolds **(c)** Live/Dead fluorescence staining **(d)** standardized DNA content of MSCs grown with rGO/SF scaffolds for 1 day, 4 weeks, and 8 weeks. In vitro osteogenic development of MSCs: gene expression levels of **(e)** BSP, **(f)** RUNX2, and **(g)** normalized calcium deposition of MSCs cultivated on rGO/SF scaffolds for 4 and 8 weeks. Reproduced with permission from [199]

Li et al. synthesized graphene oxide/silk fibroin (GO/SF) scaffolds using a salt-leaching technique, followed by in situ reduction with ascorbic acid to produce rGO/SF scaffolds, aimed at replicating the porosity architecture of natural bone. SEM research demonstrated densely interconnected pores both at the surface and throughout the interior, with variable pore size depending on GO content. The FTIR spectra **(**Fig. 7 (a)) exhibited characteristic silk fibroin peaks at 1235, 1530, and 3280 cm⁻¹, thereby affirming the integrity of the silk fibroin structure, while the peak at 1410 cm⁻¹ indicated the presence of reduced graphene oxide (rGO). No new chemical bonds were established; however, examination of the amide I area **(**Fig. 7 (b)) demonstrated an increase in β-sheet content from 46.36% to 51.12%, signifying improved structural stability. Mechanical testing revealed that Young’s modulus rose from 5.5 ± 0.5 to 14.6 ± 2.1 kPa with increased rGO concentration. Biological assessment demonstrated enhanced MSC adherence, proliferation, and mineralisation in rGO/SF scaffolds relative to pure SF. Live/Dead staining **(**Fig. 7 (c)) demonstrated elevated cell viability, with increasing scaffold coverage by viable cells over a period of 8 weeks. The quantification of DNA content **(**Fig. 7 (d)) established a positive correlation between proliferation and rGO concentration, with the 3% rGO/SF scaffold facilitating the maximum cell density. The osteogenic differentiation markers BSP and RUNX2 **(**Fig. 7 (e, f)) exhibited considerable upregulation in the presence of rGO, particularly in 1% and 3% scaffolds. After eight weeks, the 3% rGO/SF scaffolds demonstrated the highest expression of RUNX2. Calcium deposition study **(**Fig. 7 (g)) indicated that all scaffolds facilitated mineralisation throughout time, with the 3% rGO/SF scaffold exhibiting the highest calcium content after 8 weeks. The results showed that the integration of rGO improves β-sheet formation, mechanical stability, MSC proliferation, osteogenic gene expression, and mineral deposition, offering rGO/SF scaffolds viable options for bone tissue creation [199].

The considerable advances made in preclinical studies utilizing graphene oxide (GO) and reduced graphene oxide (rGO) makes their current technological challenges in shifting to the clinical practice of tissue engineering all the more disappointing. Limitations to scalability, as a result of batch to batch inconsistency in the degree of oxidation, lateral size, and functionalization of the graphene, impact the cost and compliance of Good Manufacturing Practice (GMP) production [200]. The absence of tailored pathways to graphene for the FDA and EMA means that researchers will be compelled to submit comprehensive and expensive studies to illuminate the challenges of chronic toxicity and other long-term safety concerns [200, 201]. The risks of long-term implantation include chronic infections, fibrosis, overstimulation of the antioxidant defense system, and incomplete biodegradation with the possibility of the material accumulating in various organs of the body, which justifies the need for extensive testing in large animal studies lasting more than six months with respect to the adoption of safety guidelines for clinical practice [202].

Therefore, low concentrations of rGO (1–3%) improve cell adhesion and osteogenesis causing differentiation in scaffolds. Responsive degradation and controlled 3D scaffold formation remain important challenges. The main focus of recent research should be aimed at developing intelligent and responsive GO scaffolds for personalised regenerative medicine and assessing their performance in vivo for extended periods.

### Graphene Oxide-Based Materials in Theranostics

Theranostics is an advanced biomedical methodology that integrates diagnostic and therapeutic functions into a single platform, enabling concurrent disease identification, targeted intervention, and real-time assessment of therapeutic efficacy. In contrast to traditional methods, which divide diagnosis and therapy, theranostic systems offer a more efficient and individualized approach to disease management. Carbon nanomaterials, including quantum dots, graphene oxide, and graphene, have been extensively investigated for tumor detection, chemotherapy, photothermal therapy, photodynamic therapy, and associated biological uses [203]. Graphene can transport anticancer drugs into cancer cells within nanoparticles [204]. The surfaces of graphene have both covalent and noncovalent interactions, which are crucial for their interactions with cancer cells, tissues, and anticancer medications. A “smart” nanomaterial, graphene may activate its therapeutic characteristics by interacting with tumor microenvironments, pH levels, and endogenous and exogenous stimuli, according to many studies [205, 206].

Two important elements that impact the dynamics of tumor microenvironments are hydrogen peroxide and acidic pH. The effects of hydrogen peroxide may be imitated by a particular type of graphene oxide, N-GO, as shown by Lin et al. [207]. One of these things that happens with HeLa cancers is that the levels of reactive oxygen species rise, and the cancer cells die off because hydrogen peroxide is converted into ROS-hydroxyl radicals (HO). Normal cells and tissues are unaffected by HO radicals, despite their harmful nature. These steps might be useful because they lessen tumor medication resistance, lessen the likelihood of side effects in healthy cells and tissues, and make different tumor therapies more biocompatible.

Because their surface properties can be tuned to be either highly hydrophobic or highly hydrophilic, depending on the amount of graphene sheets and physical or chemical conjugation with different molecules such as surfactants and polyethylene glycol, graphene-based materials are attractive for gene and drug delivery. It is possible to adjust the distribution rate to meet individual needs by functionalizing the material in a way that produces an ideal hydrophilic-hydrophobic ratio. In addition, 2D graphene materials can absorb both visible and infrared light, which may lead to photothermia. This process is mostly used to trigger delivery [181].

**Fig. 8:**
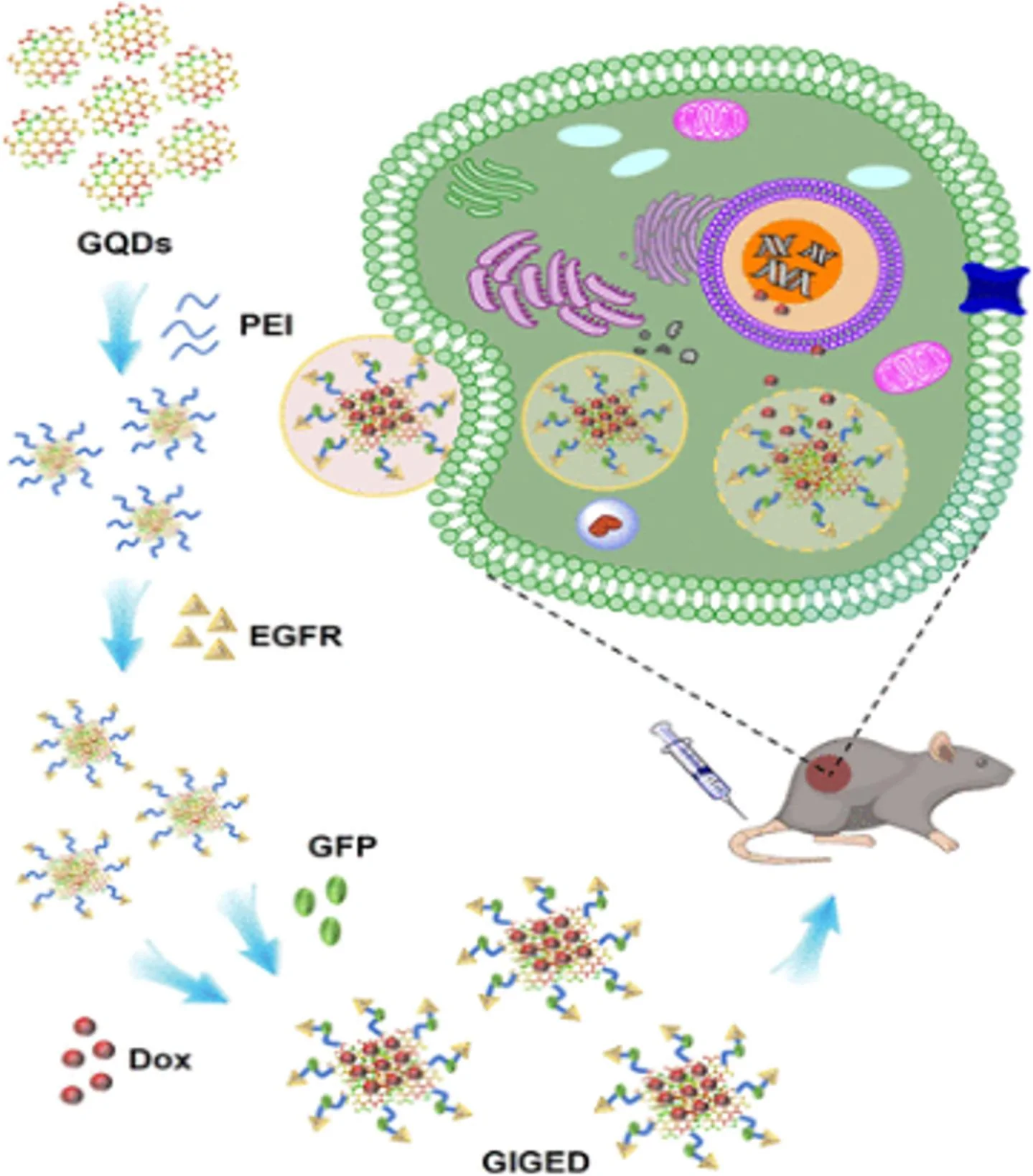
A schematic illustration of the synthesis and therapeutic pathway of a targeted drug delivery system. Reproduced with permission from [209]

Graphene is one of the nanocarriers used in gene therapy, a medical method that aims to treat or prevent disease by destroying harmful or genetically modified genes [208]. The potential eradication of colon cancer tumors in mouse models was found by Lo et al. to be facilitated by graphene quantum dots **(**Fig. 8**)** [209]. The GQDs were covalently attached to the cancer cells using polyethylenimine and green fluorescent proteins. After the tumor cell membrane was made permeable, doxorubicin was injected. Combining doxorubicin with the GQDs was more effective than using either drug alone in suppressing colon cancer tumors. When just the GQDs were present, the toxicity levels dropped.

GO-based theranostic platforms have shown strong potential by combining imaging and therapy in a single system. Žárská et al. (2024) developed PEGylated GO nanoplatforms for dual-drug delivery (cisplatin derivative + doxorubicin) and achieved high loading capacity with controlled release, demonstrating significantly enhanced anticancer activity in 3D glioblastoma and osteosarcoma models compared to single-drug carriers [210]. Similarly, Wu et al. (2025) highlighted that functionalized GO improves tumour targeting and reduces multidrug resistance through synergistic chemo-photothermal effects [211].

However, critical comparison across studies reveals important limitations. While dual-drug GO systems offer superior therapeutic outcomes over monotherapy, they still face challenges such as burst release and complex synthesis. Pristine GO shows lower photothermal conversion efficiency and higher toxicity than PEGylated or mitochondria-targeted variants [125, 212]. Most studies remain at the in vitro or small-animal stage, with very limited long-term biodistribution and scalability data. Overall, functionalized GO platforms outperform unmodified GO in loading efficiency and efficacy, but none have yet progressed to clinical trials due to regulatory and reproducibility concerns.

To determine if the toxicity of the antineoplastic medication methotrexate is enhanced in tumors, GO has been used as a nanocarrier for the drug. Using pig skin fibroblasts, hepatocellular carcinoma cells, and human embryonic kidney cells, Abdelhamid and Hussein assessed the drug in combination with graphene [213]. Results showed that HEK293A cancer cells were more hazardous than normal cells. New evidence suggests that medications naturally lean toward nanocarriers. There are a lot of drugs that form hydrogen bonds and π-π interactions with the surface of graphene [214]. Graphene oxide (GO)-based nanomaterials have distinct advantages over traditional therapeutic systems by facilitating the integration of diagnostic and therapy within just one platform, hence providing targeted drug delivery, imaging guidance, and controlled release. Their extensive surface area, adjustable surface chemistry, and excellent water stability provide substantial drug loading and straightforward functionalization with targeting or imaging agents, enhancing precision and minimizing off-target toxicity. In theranostic applications, GO composites can provide photothermal or combined chemo-photothermal therapy, enhancing treatment efficacy compared to traditional single-function systems [215].

### Infection Treatment Using Graphene-Based Materials

A thorough understanding of graphene’s processes and functions in biomedical applications requires an investigation into its antimicrobial and antibacterial characteristics. One common way to study microbial colonies is to observe their biofilm formation [216]. It is challenging to avoid microbial contamination of biofilms and other substances. The effectiveness of disease prevention and treatment is threatened by the rise of antibiotic resistance. To fight against antimicrobial (multi)resistant bacterial strains, it is crucial to find new antimicrobial medications. Materials connected to graphene might be included in such agents [217].

Ventilators, urinary catheters, and dental tools are just a few examples of the many medical devices that have been known to use various microbes. Reducing contamination levels requires the development of devices with antimicrobial coatings. Suitable nanomaterial layers or device coatings for germ elimination may be manufactured using 2D graphene oxide-based materials [218]. Studies on the microbiological activity of *E. coli* have made use of graphene and metal plates covered with GO. Graphene and GO aluminum plates had substantial antibacterial efficacy, and results showed that *E. coli* activity was suppressed. Graphene nanowalls have shown potent antibacterial properties against bacterial strains classified as Gram-positive and Gram-negative when applied as a coating on stainless steel substrates. The rough edges of the graphene nanowalls seemed to damage the bacterial membranes [219]. Yan et al. created a ternary rGO/g-C_3_N_4_/TiO_2_ nanocoating on titanium-based implants to provide photoelectric stimulation for simultaneous bone and nerve regeneration. The integration of rGO augmented charge transport, whereas g-C_3_N_4_ and TiO_2_ boosted visible-light-driven photoelectrochemical performance via effective charge carrier separation. The nanocoating, when subjected to light irradiation, enhanced the osteogenic differentiation of MC3T3-E1 cells and facilitated neurite outgrowth in PC12 cells, with the most pronounced effect noted in the rGO-containing system. In a coculture model, the augmented release of calcitonin gene-related peptide (CGRP) from neural cells further stimulated osteogenic activity, illustrating the potential of photoresponsive nanocoatings for concurrent nerve and bone regeneration [170]. Halouane et al. synthesized 2-nitrodopamine-functionalized magnetic nanoparticles immobilized on reduced graphene oxide (rGO) for the rapid and selective extraction of *Escherichia coli* from aqueous and serum specimens. The nanocomposite attained a capture effectiveness of up to 99.9% within 30 min, even at minimal bacterial concentrations (1 × 10¹ cfu mL⁻¹). Subsequent functionalization using PEG-modified pyrene units and anti-fimbrial antibodies facilitated the selective targeting of pathogenic *E. coli* strains within complicated biological environments [172]. Zeng et al. suggested a multifunctional graphene-based nanodrug delivery system for the cure of drug-resistant osteosarcoma. The device was engineered for near-infrared imaging-guided therapy, featuring preferential tumor accumulation and mitochondrial targeting capabilities. It facilitated synergistic photodynamic and photothermal therapy, which has arisen as a potential approach to surmount chemotherapy resistance in osteosarcoma by augmenting therapeutic efficacy at the cellular organelle level [15]. Shin et al. designed a graphene oxide-based fluorescence sensing system (GOViRA) for the real-time detection of intracellular dengue virus RNA in live cells. The platform comprises a dye-labeled peptide nucleic acid (PNA) probe that is complementary to the DENV genome, together with dextran-coated reduced graphene oxide, which suppresses fluorescence until hybridization with viral RNA reestablishes the signal. This technique facilitated the sensitive and quantitative detection of viral RNA and was subsequently utilized for high-throughput screening of antiviral drugs. This technique discovered ulipristal as a possible inhibitor of DENV infection, demonstrating verified antiviral efficacy both in vitro and in vivo, possibly via inhibiting viral entry into host cells. Graphene-based materials exhibit potent antimicrobial activity through membrane disruption, oxidative stress, and photothermal effects. Darini et al. (2025) reported that graphene sheets embedded with TiO_2_ and CaO nanoparticles achieved excellent microbial reduction in wastewater, lowering counts to as low as 3.0–4.85 CFU/mL for E. coli and S. aureus — significantly better than individual metal oxide components [220].

Materials based on graphene oxide (GO) reveal the potential in many areas including biocompatibility, biosensing, bioimaging, controlled delivery of drugs and genes, tissue engineering, theranostics, and antimicrobial applications. However, a critical analysis uncovers several limitations including GO applications with conflicting results, the ability to scale, and reliability. Compared to unmodified GO, functionalized and reduced GO (rGO) deliver > 90% cell viability, increased loading of therapeutic molecules (up to 65 wt% DOX), enhanced photothermal conversion, and improved targeting of tumors (e.g., rGO@AuNS hybrids give a peak PA signal sustained for > 4 h vs. GO that was not reduced). In the field of biomedicine, comparative studies suggest CHAIE–GO systems yield the lowest limit of detection (LOD) for miRNA at 0.42 pM, the highest level of osteogenic marker upregulation and > 95% wound closure achieved in 15 days was noted for rGO/silk fibroin scaffolds, and the highest capture of E. coli was found for magnetic rGO hybrids (99.9% efficiency).

On the negative side of the cytotoxicity, oxidative stress, and long-term biodistribution studies (contradictory findings for renal clearance vs. accumulation in the liver/spleen) pose major concerns. Variability of oxidation degree, a complex multi-step synthesis, and limited GMP-compliant scalability pose problems for clinical use. GO-based hybrid systems (PEGylated GO, GO–Fe₃O₄, β-CD-GO@MIL-100(Fe)) present the best overall trade-offs for controlled drug delivery, imaging contrast, and mechanical reinforcement. The majority of studies along these lines lack consistent large-scale validation. The most pressing concern for the field of biomedicine is to design production methodologies that are cost-effective in order to validate the results of laboratory.

## Conclusion

Drug delivery, bioimaging, and tissue engineering are just a few of the many biomedical domains that have found uses for it due to its mechanical strength, large surface area, and adaptable functional groups like hydroxyl, carboxyl, and epoxy groups. Recent advancements in the functionalization of GO have addressed its previously observed limitations, such as poor solubility and potential cytotoxicity. The reduction of GO to form reduced graphene oxide through non-toxic reducing agents has significantly improved its dispersion in aqueous environments, enhancing its biocompatibility without compromising its structural integrity. GO can interact with biomolecules to improve targeted medication delivery and gene therapy to overcome the constraints of standard drug administration methods. The ability of GO to degrade safely within the human body eliminates concerns regarding the accumulation of non-biodegradable materials, making it suitable for applications such as wound healing and the treatment of chronic conditions, including neurodegenerative diseases like Alzheimer’s. When it comes to smart drug delivery systems, GO has played a key role in enhancing the regulated release of medicinal drugs. Conjugating GO with targeting molecules takes advantage of its surface functional groups, allowing for the targeted delivery of medications to damaged cells, which is a crucial aspect of cancer therapy. Improved therapeutic efficiency and reduced systemic toxicity have been achieved by controlling the release of medications from GO-based carriers, which is affected by environmental factors, including pH. Their application in drug delivery and gene therapy exhibited encouraging outcomes via regulated release mechanisms, high loading efficiency, and targeted distribution. Moreover, their integration into tissue engineering scaffolds enhances mechanical stability, cellular adhesion, proliferation, and regeneration capacity. The antibacterial and antimicrobial properties of these materials render them interesting options for infection-resistant biomedical equipment and coatings.

This review does a great job at demonstrating the potential of GO based nanomaterials for the many branches of medical application. There are, however, some things that are still being developed that we might not be able to account for. Because this is a research review, and not a systematic review, the articles that cited GO based nanomaterials were selectively chosen. This has potential bias. Since this field is growing quickly, we considered literature from early 2024 to 2026, but we are likely missing some recent examples. The majority of research done in this field remains at the design stage, including small and large scale research, and we only have limited first-in-human safety studies. For that reason, we are not able to contribute to the safety or efficacy of this research or predict how it would perform in a real world application. Finally, we are focusing primarily on GO/rGO platforms and we are not including a deep analysis of nanocarriers, especially liposomes, silica nanoparticles, or metallic nanoparticles in this review. These examples show that more systematic reviews and research studies are needed.

## Future Aspects

The future of GO in biomedical applications hinges on several critical areas of development:

1. Although significant efforts have been made in reducing the cytotoxicity of GO, further optimization of its biocompatibility remains a priority. Future research should focus on refining the surface functionalization of GO to achieve minimal immunogenicity and long-term biocompatibility, particularly for in vivo applications.
2. Subsequent research must prioritize elucidating dose-dependent toxicity, biodistribution, immune system activation, degradation kinetics, organ accumulation, and clearance mechanisms in therapeutically applicable contexts.
3. The integration of GO in DDS can be further improved by incorporating smart release mechanisms, such as stimuli-responsive systems that respond to changes in pH, temperature, or enzymatic activity. This approach would allow for highly specific, controlled, and sustained drug release, enhancing therapeutic efficacy while minimizing side effects. Additionally, combining GO with other materials like polymers, liposomes, and nanocarriers could yield hybrid systems with synergistic effects, further advancing the potential for personalized medicine.
4. The incorporation of GO with other imaging agents could improve spatial resolution and tissue penetration, enabling non-invasive in vivo monitoring of disease progression and therapeutic response.
5. A combination of GO with photothermal or photodynamic therapies could offer a powerful tool for treating tumors with high precision while minimizing systemic toxicity.
6. GO’s potential in tissue engineering, particularly in bone and neural regeneration, remains largely unexplored. Future work could focus on developing GO-based scaffolds that promote cell proliferation, differentiation, and tissue integration. By combining GO with growth factors, cytokines, or other biomolecules, it may be possible to create functional tissue constructs for the regeneration of damaged organs and tissues.
7. Clinical translation necessitates enhanced alignment with regulatory frameworks set by entities such as the U.S. Food and Drug Administration and the European Medicines Agency. Subsequent research must include standardized toxicity evaluation methodologies, synthesis methods compliant with Good Manufacturing Practices, validation of sterilizing processes, and testing for shelf-life stability.

In conclusion, while graphene oxide has shown immense promise in the biomedical field, continued research and development are essential to overcoming the challenges related to its toxicity, stability, and functionalization. The future of GO in medicine will likely depend on its integration with other cutting-edge technologies, such as artificial intelligence, nanorobotics, and personalized medicine, to further enhance its capabilities in treating complex diseases.

## Data Availability

No data was used for this research.

## References

[1] Wadan, A. H. S., et al. (2025). Mitochondrial-based therapies for neurodegenerative diseases: a review of the current literature . *Naunyn-Schmiedeberg’s Archives of Pharmacology*, (pp. 1–30) 10.1007/s00210-025-04014-0 PMC12449425 40163151

[2] Gupta, M. N. (2025). Importance of marker molecules in designing sensors and theranostic devices for chronic wound care. *Molecular Pharmaceutics.* 10.1021/acs.molpharmaceut.5c00282 40608299

[3] Yao, Y., et al. (2020). Nanoparticle-based drug delivery in cancer therapy and its role in overcoming drug resistance. *Frontiers in molecular biosciences*, *7*, 193. 10.3389/fmolb.2020.00193 PMC7468194 32974385

[4] Pratap, A., et al. (2024). Association of sociodemographic with epidemiological profile of chronic wound patients attending a tertiary care hospital. *Indian Journal of Preventive & Social Medicine*, *55*(3), 196–204.

[5] Iyun, A. O., et al. (2024). The incidence and prevalence of chronic wounds from a major plastic surgery service from a metropolitan city in south western Nigeria: A sixteen-year retrospective review. *Journal of Tissue Viability*, *33*(4), 877–882. 10.1016/j.jtv.2024.08.005 39181782

[6] Muzammal, M., et al. (2024). Graphene-based nanomaterials are a potent and innovative technology for the treatment of cancer: A review. *ChemistrySelect*, *9*(40), e202403554.

[7] Sontakke, A. D., Tiwari, S., & Purkait, M. K. (2024). *Surface modifications of graphene oxide nanomaterials for analytical applications, in Comprehensive Analytical Chemistry* (pp. 1–37). Elsevier.

[8] Li, S., et al. (2018). Surface modification and thermal performance of a graphene oxide/novolac epoxy composite. *RSC advances*, *8*(37), 20505–20516. 10.1039/c8ra02847h PMC9080807 35542377

[9] Karimi, M., & Grayeli, A. (2024). *Next-generation graphene-based photocatalysts for purification of air and water, in Graphene-Based Photocatalysts for Hydrogen Production and Environmental Remediation* (pp. 403–425). Springer.

[10] Tazeen, Z., et al. (2026). A decade of breakthroughs: MOF-graphene oxide catalysts for water splitting efficiency. *Reviews in Inorganic Chemistry*, *46*(1), 87–107.

[11] Javed, M., et al. (2022). Synergistic influences of doping techniques and well-defined heterointerface formation to improve the photocatalytic ability of the S‐ZnO/GO nanocomposite. *ChemistrySelect*, *7*(29), e202201913.

[12] Sontakke, A. D., Tiwari, S., & Purkait, M. K. (2023). A comprehensive review on graphene oxide-based nanocarriers: Synthesis, functionalization and biomedical applications. *FlatChem*, *38*, 100484.

[13] Kumar, R., et al. (2023). Graphene-based materials for biotechnological and biomedical applications: Drug delivery, bioimaging and biosensing. *Materials Today Chemistry.*

[14] Ali, R., et al. (2022). Graphene oxide/zinc ferrite nanocomposite loaded with doxorubicin as a potential theranostic mediu in cancer therapy and magnetic resonance imaging. *Ceramics International*, *48*(8), 10741–10750.

[15] Zeng, W. N., et al. (2021). Mitochondria-targeting graphene oxide nanocomposites for fluorescence imaging-guided synergistic phototherapy of drug-resistant osteosarcoma. *Journal of nanobiotechnology*, *19*(1), 79. 10.1186/s12951-021-00831-6 PMC7980640 33740998

[16] Sundeep, D., & Varadharaj, E. (2024). *Development of Biocompatible and Water-Soluble Reduced Graphene Oxide Using Non-Toxic Reducing Agents for Biomedical Applications*. ECS Meeting Abstracts.

[17] KurapatiCnrs, R. (2023). *Graphene oxide biodegrades with help of human enzymes.*

[18] Rhazouani, A., et al. (2023). Sub-acute toxicity of graphene oxide (GO) nanoparticles in male mice after intraperitoneal injection: Behavioral study and histopathological evaluation. *Food and chemical toxicology: an international journal published for the British Industrial Biological Research Association*, *171*, 113553. 10.1016/j.fct.2022.113553 36521574

[19] Biswas, H. S., Kundu, A. K., & Maiti, D. K. (2026). Graphene oxide nanotechnology for next-generation cancer theranostics: Merging diagnostics and therapy for precision oncology. *Applications of Nanomaterials for Oncology*, (pp. 61–94)

[20] Kazankapova, M., et al. (2025). Potential use of graphene-like nanomaterials in soil sensors and moisture monitoring. *International Journal of Agriculture and Biosciences*, *14*(4), 721–730.

[21] Mkhoyan, K. A., et al. (2009). Atomic and electronic structure of graphene-oxide. *Nano letters*, *9*(3), 1058–1063. 10.1021/nl8034256 19199476

[22] Lerf, A., et al. (1998). Structure of graphite oxide revisited. *The Journal of Physical Chemistry B*, *102*(23), 4477–4482.

[23] Erickson, K., et al. (2010). Determination of the local chemical structure of graphene oxide and reduced graphene oxide. *Advanced materials*, *22*(40), 4467–4472. 10.1002/adma.201000732 20717985

[24] Sofer, Z., et al. (2014). Neutron diffraction as a precise and reliable method for obtaining structural properties of bulk quantities of graphene. *Nanoscale*, *6*(21), 13082–13089. 10.1039/c4nr04644g 25247281

[25] Wilson, N. R., et al. (2009). Graphene oxide: structural analysis and application as a highly transparent support for electron microscopy. *ACS nano*, *3*(9), 2547–2556. 10.1021/nn900694t 19689122

[26] Hofmann, U., & Holst, R. (1939). Über die Säurenatur und die Methylierung von Graphitoxyd. *Berichte der deutschen chemischen Gesellschaft (A and B Series)*, *72*(4), 754–771.

[27] Ruess, G. (1947). Über das graphitoxyhydroxyd (graphitoxyd). *Monatshefte für Chemie und verwandte Teile anderer Wissenschaften*, *76*, p381–417.

[28] Scholz, W., & Boehm, H. (1969). Untersuchungen am graphitoxid. VI. Betrachtungen zur struktur des graphitoxids. *Zeitschrift für anorganische und allgemeine Chemie*, *369*(3-6), 327–340.

[29] Nakajima, T., & Matsuo, Y. (1994). Formation process and structure of graphite oxide. *Carbon*, *32*(3), 469–475.

[30] Dimiev, A. M., Alemany, L. B., & Tour, J. M. (2013). Graphene oxide. Origin of acidity, its instability in water, and a new dynamic structural model. *ACS nano*, *7*(1), 576–588. 10.1021/nn3047378 23215236

[31] Liu, Z., et al. (2018). Direct observation of oxygen configuration on individual graphene oxide sheets. *Carbon*, *127*, 141–148.

[32] Sun, L. (2019). Structure and synthesis of graphene oxide. *Chinese Journal of Chemical Engineering*, *27*(10), 2251–2260.

[33] Velázquez, L. D. D. M. M. (2020). MM Maestro A.Understanding the role of oxidative debris on the structure of graphene oxide films at the air-water interface: A neutron reflectivity study. *ACS Appl Mater Interfaces*, *12*(22), 25453–25463. 10.1021/acsami.0c05649 32394699

[34] Sofer, Z., et al. (2014). Highly hydrogenated graphene via active hydrogen reduction of graphene oxide in the aqueous phase at room temperature. *Nanoscale*, *6*(4), 2153–2160. 10.1039/c3nr05407a 24366534

[35] Faria, A. F., et al. (2012). Unveiling the role of oxidation debris on the surface chemistry of graphene through the anchoring of Ag nanoparticles. *Chemistry of Materials*, *24*(21), 4080–4087.

[36] Wu, Z., PittmanJr, C. U., & Gardner, S. D. (1995). Nitric acid oxidation of carbon fibers and the effects of subsequent treatment in refluxing aqueous NaOH. *Carbon*, *33*(5), 597–605.

[37] Bonanni, A., et al. (2014). Oxidation debris in graphene oxide is responsible for its inherent electroactivity. *ACS nano*, *8*(5), 4197–4204. 10.1021/nn404255q 24783949

[38] Claramunt, S., et al. (2015). The importance of interbands on the interpretation of the Raman spectrum of graphene oxide. *The Journal of Physical Chemistry C*, *119*(18), 10123–10129.

[39] Thirupathi, R., et al. (2014). Organic fragments from graphene oxide: Isolation, characterization and solvent effects. *Journal of Chemical Sciences*, *126*, 541–545.

[40] Rourke, J. P., et al. (2011). The real graphene oxide revealed: stripping the oxidative debris from the graphene-like sheets. *Angewandte Chemie International Edition*, *50*(14), 3173–3177. 10.1002/anie.201007520 21432951

[41] Kim, J. (2021). Tuning the hierarchical pore structure of graphene oxide through dual thermal activation for high-performance supercapacitor. *Scientific Reports*, *11*(1), 2063. 10.1038/s41598-021-81759-7 PMC7822934 33483594

[42] Zhang, Z., Schniepp, H. C., & Adamson, D. H. (2019). Characterization of graphene oxide: Variations in reported approaches. *Carbon*, *154*, 510–521.

[43] Chuah, R., et al. (2020). Synthesis and characterization of reduced graphene oxide using the aqueous extract of Eclipta prostrata. *Biotech*, *3*(8), 364. 10.1007/s13205-020-02365-4 PMC7394997 32832325

[44] Jiao, X., et al. (2017). Comparison of the characteristic properties of reduced graphene oxides synthesized from natural graphites with different graphitization degrees. *RSC advances*, *7*(82), 52337–52344.

[45] Tshangana, C., Muleja, A., & Mamba, B. (2022). Photocatalytic activity of graphene oxide quantum dots in an effluent from a South African wastewater treatment plant. *Journal of Nanoparticle Research*, *24*(2), 43.

[46] Karimi, B., & Ramezanzadeh, B. (2017). A comparative study on the effects of ultrathin luminescent graphene oxide quantum dot (GOQD) and graphene oxide (GO) nanosheets on the interfacial interactions and mechanical properties of an epoxy composite. *Journal of colloid and interface science*, *493*, 62–76. 10.1016/j.jcis.2017.01.013 28088122

[47] HummersJr, W. S., & Offeman, R. E. (1958). Preparation of graphitic oxide. *Journal of the american chemical society*, *80*(6), 1339–1339.

[48] Kovtyukhova, N. I., et al. (1999). Layer-by-layer assembly of ultrathin composite films from micron-sized graphite oxide sheets and polycations. *Chemistry of materials*, *11*(3), 771–778.

[49] Eigler, S. (2015). Graphite sulphate–a precursor to graphene. *Chemical Communications*, *51*(15), 3162–3165. 10.1039/c4cc09381j 25605427

[50] Yu, H., et al. (2016). High-efficient synthesis of graphene oxide based on improved hummers method. *Scientific Reports*, *6*(1), 36143. 10.1038/srep36143 PMC5093679 27808164

[51] Eigler, S., et al. (2013). Wet chemical synthesis of graphene. *Advanced materials*, *25*(26), 3583–3587. 10.1002/adma.201300155 23703794

[52] Chen, H., et al. (2019). Efficient room-temperature production of high-quality graphene by introducing removable oxygen functional groups to the precursor. *Chemical Science*, *10*(4), 1244–1253. 10.1039/c8sc03695k PMC6349015 30774925

[53] Barroso-Bujans, F., et al. (2012). Tunable uptake of poly(ethylene oxide) by graphite-oxide-based materials. *Carbon, 50*, 5232–5241.

[54] Hussein, A., Sarkar, S., & Kim, B. (2016). Low temperature reduction of graphene oxide using hot-plate for nanocomposites applications. *Journal of Materials Science & Technology, 32*.

[55] Bukovska, H., et al. (2023). Evaluation and optimization of tour method for synthesis of graphite oxide with high specific surface area. *C, 9*(3), 65.

[56] Brodie, B. (1855). Note sur un nouveau procédé pour la purification et la désagrégation du graphite. *Annales de chimie et de physique, 45*, 351–353.

[57] Brodie, B. C., XIII. (1859). On the atomic weight of graphite. *Philosophical transactions of the Royal Society of London*, (149), 249–259.

[58] Staudenmaier, L. (1898). Verfahren zur darstellung der graphitsäure. *Berichte der deutschen chemischen Gesellschaft, 31*(2), 1481–1487.

[59] Jankovský, O., et al. (2017). Concentration of nitric acid strongly influences chemical composition of graphite oxide. *Chemistry–A European Journal, 23*(26), 6432–6440. 10.1002/chem.201700809 28244151

[60] Marcano, D.C., et al. (2010). Improved synthesis of graphene oxide. *ACS nano, 4*(8), 4806–4814. 10.1021/nn1006368 20731455

[61] Wojtoniszak, M., & Mijowska, E. (2012). Controlled oxidation of graphite to graphene oxide with novel oxidants in a bulk scale. *Journal of Nanoparticle Research, 14*, 1–7. 10.1007/s11051-012-1248-z PMC3501180 23193373

[62] Dreyer, D. R., et al. (2010). The chemistry of graphene oxide. *Chemical Society Reviews, 39*(1), 228–240. 10.1039/b917103g 20023850

[63] Peng, L., et al. (2015). An iron-based green approach to 1-h production of single-layer graphene oxide. *Nature communications, 6*(1), 5716. 10.1038/ncomms6716 PMC4354147 25607686

[64] Sofer, Z., et al. (2016). Innenrücktitelbild: Synthesis of Graphene Oxide by Oxidation of Graphite with Ferrate (VI) Compounds: Myth or Reality?. *Angewandte Chemie, 128*(39), 12289–12289. 10.1002/anie.201603496 27457677

[65] Yu, C., Wang, C. F., & Chen, S. (2016). Facile access to graphene oxide from ferro-induced oxidation. *Scientific reports, 6*(1), 17071. 10.1038/srep17071 PMC4730196 26818784

[66] Shen, J., et al. (2009). Fast and facile preparation of graphene oxide and reduced graphene oxide nanoplatelets. *Chemistry of materials, 21*(15), 3514–3520.

[67] Jankovský, O., et al. (2017). Fast synthesis of highly oxidized graphene oxide. *Chemistry Select, 2*(28), 9000–9006.

[68] Yu, P., et al. (2016). Mechanically-assisted electrochemical production of graphene oxide. *Chemistry of Materials, 28*(22), 8429–8438.

[69] Pei, S., et al. (2018). Green synthesis of graphene oxide by seconds timescale water electrolytic oxidation. *Nature communications, 9*(1), 145. 10.1038/s41467-017-02479-z PMC5762692 29321501

[70] Han, S., et al. (2019). The application of graphene-based biomaterials in biomedicine. *American journal of translational research, 11*(6), 3246. PMC6614642 31312342

[71] Novoselov, K. S., et al. (2012). A roadmap for graphene. *Nature, 490*(7419), 192–200. 10.1038/nature11458 23060189

[72] Dikin, D. A., et al. (2007). Preparation and characterization of graphene oxide paper. *Nature, 448*(7152), 457–460. 10.1038/nature06016 17653188

[73] Xu, X., Zhang, Z., & Yao, W. (2021). Mechanical properties of graphene oxide coupled by multi-physical field: Grain boundaries and functional groups. *Crystals, 11*(1), 62.

[74] Hu, D., et al. (2017). Strong graphene-interlayered carbon nanotube films with high thermal conductivity. *Carbon, 118*, 659–665.

[75] Shen, Y., et al. (2012). Chemical and thermal reduction of graphene oxide and its electrically conductive polylactic acid nanocomposites. *Composites Science and Technology, 72*, 1430–1435.

[76] Bellier, N., et al. (2022). Recent biomedical advancements in graphene oxide- and reduced graphene oxide-based nanocomposite nanocarriers. *Biomaterials Research, 26*(1), 65. 10.1186/s40824-022-00313-2 PMC9701399 36435846

[77] Shi, G., et al. (2018). Graphene platelets and their polymer composites: fabrication, structure, properties, and applications. *Advanced Functional Materials, 28*(19), 1706705.

[78] Du, J., & Cheng, H. M. (2012). The fabrication, properties, and uses of graphene/polymer composites. *Macromolecular Chemistry and Physics, 213*(10–11), 1060–1077.

[79] Ferrari, A. C., et al. (2015). Science and technology roadmap for graphene, related two-dimensional crystals, and hybrid systems. *Nanoscale, 7*(11), 4598–4810. 10.1039/c4nr01600a 25707682

[80] Bai, J., & Allaoui, A. (2003). Effect of the length and the aggregate size of MWNTs on the improvement efficiency of the mechanical and electrical properties of nanocomposites—experimental investigation. *Composites Part A: Applied Science and Manufacturing, 34*(8), 689–694.

[81] Bao, C., et al. (2011). Poly (vinyl alcohol) nanocomposites based on graphene and graphite oxide: a comparative investigation of property and mechanism. *Journal of Materials Chemistry, 21*(36), 13942–13950.

[82] Wang, J., et al. (2011). Preparation and mechanical and electrical properties of graphene nanosheets–poly (methyl methacrylate) nanocomposites via in situ suspension polymerization. *Journal of Applied Polymer Science, 122*(3), 1866–1871.

[83] Ramanathan, T., et al. (2008). Functionalized graphene sheets for polymer nanocomposites. *Nature nanotechnology, 3*(6), 327–331. 10.1038/nnano.2008.96 18654541

[84] Adhikari, M., Orasugh, J., & Chattopadhyay, D. (2022). *Biomedical application of polymer-graphene composites, in polymer nanocomposites containing graphene*. Elsevier, Woodhead Publishing Limited Cambridge.

[85] Zhu, Y., et al. (2010). Graphene and graphene oxide: synthesis, properties, and applications. *Advanced Materials, 22*(35), 3906–3924. 10.1002/adma.201001068 20706983

[86] Phiri, J., et al. (2018). A comparative study of mechanical, thermal and electrical properties of graphene-, graphene oxide-and reduced graphene oxide-doped microfibrillated cellulose nanocomposites. *Composites Part B: Engineering, 147*, 104–113.

[87] Yu, A., et al. (2007). Graphite nanoplatelet− epoxy composite thermal interface materials. *The Journal of Physical Chemistry C, 111*(21), 7565–7569.

[88] Guo, Y., et al. (2011). In situ polymerization of graphene, graphite oxide, and functionalized graphite oxide into epoxy resin and comparison study of on-the-flame behavior. *Industrial & Engineering Chemistry Research, 50*(13), 7772–7783.

[89] Fang, M., et al. (2010). Single-layer graphene nanosheets with controlled grafting of polymer chains. *Journal of Materials Chemistry, 20*(10), 1982–1992.

[90] Liang, J., et al. (2009). Molecular‐level dispersion of graphene into poly (vinyl alcohol) and effective reinforcement of their nanocomposites. *Advanced Functional Materials, 19*(14), 2297–2302.

[91] Kim, I. H., & Jeong, Y. G. (2010). Polylactide/exfoliated graphite nanocomposites with enhanced thermal stability, mechanical modulus, and electrical conductivity. *Journal of Polymer Science Part B: Polymer Physics, 48*(8), 850–858.

[92] Hu, Y., et al. (2010). Comparison of the thermal properties between composites reinforced by raw and amino-functionalized carbon materials. *Composites Science and Technology, 70*(15), 2176–2182.

[93] Bui, K., et al. (2011). Effective heat transfer properties of graphene sheet nanocomposites and comparison to carbon nanotube nanocomposites. *The Journal of Physical Chemistry C, 115*(10), 3872–3880.

[94] Kim, H. W., et al. (2013). Selective gas transport through few-layered graphene and graphene oxide membranes. *Science, 342*(6154), 91–95. 10.1126/science.1236098 24092738

[95] Wang, J., et al. (2011). Preparation of graphene/poly (vinyl alcohol) nanocomposites with enhanced mechanical properties and water resistance. *Polymer International, 60*(5), 816–822.

[96] Kim, H. M., Lee, J. K., & Lee, H. S. (2011). Transparent and high gas barrier films based on poly (vinyl alcohol)/graphene oxide composites. *Thin Solid Films, 519*(22), 7766–7771.

[97] Kim, H., Miura, Y., & Macosko, C. W. (2010). Graphene/polyurethane nanocomposites for improved gas barrier and electrical conductivity. *Chemistry of materials, 22*(11), 3441–3450.

[98] Kim, H., & Macosko, C. W. (2009). Processing-property relationships of polycarbonate/graphene composites. *Polymer, 50*(15), 3797–3809.

[99] Tadyszak, K., Wychowaniec, J. K. & Litowczenko, J. (2018). Biomedical applications of graphene-based structures. *Nanomaterials, 8*(11), 944. 10.3390/nano8110944 PMC6267346 30453490

[100] Bai, S., et al. (2018). Enhancement of mechanical and electrical properties of graphene/cement composite due to improved dispersion of graphene by addition of silica fume. *Construction and Building Materials, 164*, 433–441.

[101] Cusati, T., et al. (2017). Electrical properties of graphene-metal contacts. *Scientific Reports, 7*(1), 5109. 10.1038/s41598-017-05069-7 PMC5506027 28698652

[102] Nimbalkar, A., & Kim, H. (2020). Opportunities and challenges in twisted bilayer graphene: a review. *Nano-Micro Letters, 12*, 1–20. 10.1007/s40820-020-00464-8 PMC7770697 34138115

[103] Pham, V.H., et al. (2011). Superior conductive polystyrene–chemically converted graphene nanocomposite. *Journal of Materials Chemistry, 21*(30), 11312–11316.

[104] Raghu, A. V., et al. (2008). Preparation and physical properties of waterborne polyurethane/functionalized graphene sheet nanocomposites. *Macromolecular Chemistry and Physics, 209*(24), 2487–2493.

[105] Du, J., et al. (2011). Comparison of electrical properties between multi-walled carbon nanotube and graphene nanosheet/high density polyethylene composites with a segregated network structure. *Carbon, 49*(4), 1094–1100.

[106] Stankovich, S., et al. (2006). Graphene-based composite materials. *Nature, 442*(7100), 282–286. 10.1038/nature04969 16855586

[107] Aydin, T., et al. (2018). Graphene based materials in neural tissue regeneration. *Cell Biology and Translational Medicine, Volume 3: Stem Cells, Bio-materials and Tissue Engineering*, 129–142. 10.1007/5584_2018_221 29882208

[108] Xia, M. -Y., et al. (2019). Graphene-based nanomaterials: the promising active agents for antibiotics-independent antibacterial applications. *Journal of Controlled Release, 307*, 16–31. 10.1016/j.jconrel.2019.06.011 31185232

[109] Zare, P., et al. (2021). Graphene oxide: opportunities and challenges in biomedicine. *Nanomaterials, 11*(5), 1083. 10.3390/nano11051083 PMC8143506 33922153

[110] Fatima, N., et al. (2021). Recent developments for antimicrobial applications of graphene-based polymeric composites: A review. *Journal of Industrial and Engineering Chemistry, 100*, 40–58.

[111] Wan, C., & Chen, B. (2011). Poly (ε-caprolactone)/graphene oxide biocomposites: mechanical properties and bioactivity. *Biomedical Materials, 6*(5), 055010. 10.1088/1748-6041/6/5/055010 21921319

[112] Raman, A. P. S., Jain, P., & Singh, P. (2024). Graphene-based photocatalysts for biomedical applications and eco-friendly biomedical waste management. *In graphene-based photocatalysts for hydrogen production and environmental remediation *(p. 603–619). Springer.

[113] Nicolussi, P., et al. (2025). Biocompatibility of water-dispersible pristine graphene and graphene oxide using a close-to-human animal model: A pilot study on swine. *Advanced Healthcare Materials, 14*(10), 2401783. 10.1002/adhm.202401783 PMC12004448 39385652

[114] Xu, M., et al. (2016). Improved *in vitro and in vivo biocompatibility of graphene oxide through surface modification*: Poly(acrylic acid)-functionalization is superior to pegylation. *ACS Nano, 10*(3), 3267–3281. 10.1021/acsnano.6b00539 26855010

[115] Ayreen, Z., et al. (2024). Perilous paradigm of graphene oxide and its derivatives in biomedical applications: Insight to immunocompatibility. *Biomedicine & Pharmacotherapy, 176*, 116842. 10.1016/j.biopha.2024.116842 38810404

[116] Shao, Y., et al. (2015). Graphene-based materials for flexible supercapacitors. *Chemical Society Reviews, 44*(11), 3639–3665. 10.1039/c4cs00316k 25898904

[117] Ghulam, A.N., et al. (2022). Graphene oxide (GO) materials—applications and toxicity on living organisms and environment. *Journal of Functional Biomaterials, 13*(2), 77. 10.3390/jfb13020077 PMC9224660 35735932

[118] Gosai, A., et al. (2021). Application of functionalized graphene oxide based biosensors for health monitoring: Simple graphene derivatives to 3D printed platforms. *Biosensors, 11*(10), 384. 10.3390/bios11100384 PMC8533804 34677340

[119] Mshelia, S. S., Jain, P., & Gaur, M. (2025). Graphene oxide-based smart sensors: Synthesis, functionalization strategies, and biosensing applications. *Nano-Structures & Nano-Objects, 43*, 101532.

[120] Kostadinova, T., et al. (2021). Effect of graphene characteristics on morphology and performance of composite noble metal-reduced graphene oxide SERS substrate. *Molecules, 26*(16), 4775. 10.3390/molecules26164775 PMC8401241 34443368

[121] Hussain, S., & Maktedar, S. S. (2023). Structural, functional and mechanical performance of advanced Graphene-based composite hydrogels. *Results in Chemistry, 6*, 101029.

[122] Raman, A. P. S., et al. (2024). Composite nanoarchitectonics based on graphene oxide in energy storage and conversion: status, challenges & opportunities. *Journal of Inorganic and Organometallic Polymers and Materials, 34*(11), 5035–5065.

[123] Andrews, J. P. M., et al. (2024). First-in-human controlled inhalation of thin graphene oxide nanosheets to study acute cardiorespiratory responses. *Nature Nanotechnology, 19*(5), 705–714. 10.1038/s41565-023-01572-3 PMC11106005 38366225

[124] INBRAIN Neuroelectronics. (2025). *Interim analysis of first-in-human study of graphene-based brain-computer interface*.

[125] Huang, X., et al. (2025) Graphene oxide: Preparation and medical research. *Materials, 18*(12), 2855. 10.3390/ma18122855 PMC12195008 40572985

[126] Suarez-Merino, B., et al. (2025). Regulatory challenges and risk assessment of graphene-enabled products: insights for safe commercialisation in Europe. *2D Materials, 12*(4), 043001.

[127] Devi, K. S., Prakash, J., & Tsujimura, S. (2024). Graphene oxide-based nanomaterials for electrochemical bio/immune sensing and its advancements in health care applications: A review. *Hybrid Advances, 5*, 100123.

[128] Lee, J., et al. (2016). Biosensors based on graphene oxide and its biomedical application. *Advanced Drug Delivery Reviews, 105*, 275–287. 10.1016/j.addr.2016.06.001 PMC7102652 27302607

[129] Tahir, S., et al. (2025). A comprehensive review on functionalized graphene oxide nanoparticles for fluorescence properties and potential in biosensing and water remediation. *Chemistry Select, 10*(29), e00220.

[130] Hasler, R., et al. (2024). “Clickable” graphene nanoribbons for biosensor interfaces. *Nanoscale Horizons, 9*(4), 598–608. 10.1039/d3nh00590a PMC10962640 38385442

[131] Khan, A., DeVoe, E., & Andreescu, S. (2023). Carbon-based electrochemical biosensors as diagnostic platforms for connected decentralized healthcare. *Sensors & Diagnostics, 2*(3), 529–558.

[132] Menaa, F., et al. (2021). Graphene, an interesting nanocarbon allotrope for biosensing applications: advances, insights, and prospects. *Biomedical Engineering and Computational Biology, 12*, 1179597220983821. 10.1177/1179597220983821 PMC7917420 33716517

[133] Liu, L., et al. (2022). Thrombin determination using graphene oxide sensors with co-assisted amplification. *Micromachines, 13*(9), 1435. 10.3390/mi13091435 PMC9502102 36144058

[134] Gao, L., et al. (2015). Highly sensitive detection for proteins using graphene oxide-aptamer based sensors. *Nanoscale, 7*(25), 10903–10907. 10.1039/c5nr01187f 25939390

[135] Huang, A., et al. (2017). Quantitative fluorescence quenching on antibody-conjugated graphene oxide as a platform for protein sensing. *Scientific Reports, 7*(1), 40772. 10.1038/srep40772 PMC5233999 28084438

[136] Simsikova, M. (2017). Interaction of graphene oxide with proteins and applications of their conjugates. *Journal of Nanomedicine Research, 5*(2), 00109.

[137] Al-Salman, H., et al. (2024). Graphene oxide-based biosensors for detection of lung cancer: A review. *Results in Chemistry, 7*, 101300.

[138] Pan, X., et al. (2019). A graphene oxide-gold nanostar hybrid based-paper biosensor for label-free SERS detection of serum bilirubin for diagnosis of jaundice. *Biosensors and Bioelectronics, 145*, 111713. 10.1016/j.bios.2019.111713 31542676

[139] Sadeghi, Z., Shojaeihagh, N., & Shirkani, H. (2021). Multiple-step graphene grating optical sensors based on surface plasmons in IR range for ultra-sensing biomolecules. *Materials Science and Engineering: B, 265*, 114988.

[140] Meng, F., et al. (2018). One-step synthesis of Au/SnO2/RGO nanocomposites and their VOC sensing properties. *IEEE Transactions on Nanotechnology, 17*(2), 212–219.

[141] Song, X., et al. (2019). Graphene‐oxide‐modified lanthanide nanoprobes for tumor‐targeted visible/NIR‐II luminescence imaging. *Angewandte Chemie International Edition, 58*(52), 18981–18986. 10.1002/anie.201909416 31603623

[142] Mishra, S. R., et al. (2024). Biocompatible fluorescent graphene oxide quantum dots for imaging of drosophila melanogaster. *ACS omega, 9*(37), 38916–38924. 10.1021/acsomega.4c05244 PMC11411514 39310168

[143] Zheng, P., & Wu, N. (2017). Fluorescence and sensing applications of graphene oxide and graphene quantum dots: A review. *Chemistry–An Asian Journal, 12*(18), 2343–2353. 10.1002/asia.201700814 PMC5915373 28742956

[144] Gonzalez-Rodriguez, R., Campbell, E., & Naumov, A. (2019). Multifunctional graphene oxide/iron oxide nanoparticles for magnetic targeted drug delivery dual magnetic resonance/fluorescence imaging and cancer sensing. *PLoS One, 14*(6), e0217072. 10.1371/journal.pone.0217072 PMC6553710 31170197

[145] Zhang, Y., et al. (2022). Cytotoxicity effect of iron oxide (Fe3O4)/graphene oxide (GO) nanosheets in cultured HBE cells. *Frontiers in Chemistry, 10*, 888033. 10.3389/fchem.2022.888033 PMC9124895 35615314

[146] Piché, D., et al. (2019). Targeted T 1 magnetic resonance imaging contrast enhancement with extraordinarily small CoFe2O4 nanoparticles. *ACS Applied Materials & Interfaces, 11*(7), 6724–6740. 10.1021/acsami.8b17162 PMC6385080 30688055

[147] Wang, W., et al. (2024). Utilization of nanomaterials in MRI contrast agents and their role in therapy guided by imaging. *Frontiers in Bioengineering and Biotechnology, 12*, 1484577. 10.3389/fbioe.2024.1484577 PMC11611570 39628648

[148] Song, Y. -Y., et al. (2018). Graphene oxide coating core–shell silver sulfide@ mesoporous silica for active targeted dual-mode imaging and chemo-photothermal synergistic therapy against tumors. *Journal of Materials Chemistry B, 6*(29), 4808–4820. 10.1039/c8tb00940f 32254308

[149] Lin, J., Huang, Y., & Huang, P. (2018). Graphene-based nanomaterials in bioimaging. *Biomedical Applications of Functionalized Nanomaterials*, 247–287.

[150] Jia, X., et al. (2020). Functionalized graphene@ gold nanostar/lipid for pancreatic cancer gene and photothermal synergistic therapy under photoacoustic/photothermal imaging dual‐modal guidance. *Small, 16*(39), 2003707. 10.1002/smll.202003707 32851808

[151] Zhang, Y., et al. (2022). Synthesis of liquid gallium@ reduced graphene oxide core–shell nanoparticles with enhanced photoacoustic and photothermal performance. *Journal of the American Chemical Society, 144*(15), 6779–6790. 10.1021/jacs.2c00162 35293736

[152] Yang, L., et al. (2018). A highly sensitive strategy for fluorescence imaging of microRNA in living cells and in vivo based on graphene oxide-enhanced signal molecules quenching of molecular beacon. *ACS applied materials & interfaces, 10*(8), 6982–6990. 10.1021/acsami.7b19284 29405060

[153] Song, Y., et al. (2024). Catalytic hairpin assembly-based AIEgen/graphene oxide nanocomposite for fluorescence-enhanced and high-precision spatiotemporal imaging of microRNA in living cells. *Biosensors and Bioelectronics, 259*, 116416. 10.1016/j.bios.2024.116416 38797033

[154] Su, X., et al. (2022). Enhanced photothermal and photoacoustic performance of graphene oxide in nir-ii biowindow by chemical reduction. *Photonics, 9*(1), 2.

[155] Einafshar, E., et al. (2024). Curcumin loaded β-cyclodextrin-magnetic graphene oxide nanoparticles decorated with folic acid receptors as a new theranostic agent to improve prostate cancer treatment. *Carbohydrate Polymers, 340*, 122328. 10.1016/j.carbpol.2024.122328 38857995

[156] Zarei, A., et al. (2024). Diopside/graphene-oxide nanocomposite coatings on AZ31 Mg alloy for biomedical applications: Surface characteristics, corrosion, and biocompatibility properties. *Surface and Coatings Technology, 479*, 130482.

[157] Cai, X., et al. (2019). Manganese oxide nanoparticles as MRI contrast agents in tumor multimodal imaging and therapy. *International Journal of Nanomedicine*, 8321–8344. 10.2147/IJN.S218085 PMC6814316 31695370

[158] Li, H., & Meade, T. J. (2019). Molecular magnetic resonance imaging with Gd (III)-based contrast agents: challenges and key advances. *Journal of the American Chemical Society, 141*(43), 17025–17041. 10.1021/jacs.9b09149 PMC6821590 31593630

[159] Tao, W., et al. (2023). Revealing the chemical nature of functional groups on graphene oxide by integrating potentiometric titration and ab initio calculations. *ACS omega, 8*(27), 24332–24340. 10.1021/acsomega.3c01596 PMC10339393 37457448

[160] Sadighian, S., et al. (2021). Preparation of graphene oxide/Fe3O4 nanocomposite as a potential magnetic nanocarrier and MRI contrast agent. *Chemistry Select, 6*(12), 2862–2868.

[161] Gulati, S., et al. (2022). Magnetic nanocarriers adorned on graphene: promising contrast-enhancing agents with state-of-the-art performance in magnetic resonance imaging (MRI) and theranostics. *Materials Advances, 3*(7), 2971–2989.

[162] Maturi, M., et al. (2019). Current concepts in nanostructured contrast media development for in vivo photoacoustic imaging. *Biomaterials cience, 7*(5), 1746–1775. 10.1039/c8bm01444b 30901017

[163] Sheng, Z., et al. (2013). Protein-assisted fabrication of nano-reduced graphene oxide for combined in vivo photoacoustic imaging and photothermal therapy. *Biomaterials, 34*(21), 5236–5243. 10.1016/j.biomaterials.2013.03.090 23602365

[164] Wang, Z., et al. (2020). A sandwich nanostructure of gold nanoparticle coated reduced graphene oxide for photoacoustic imaging-guided photothermal therapy in the second NIR window. *Frontiers in Bioengineering and Biotechnology, 8*, 655. 10.3389/fbioe.2020.00655 PMC7338568 32695755

[165] Hu, D., et al. (2016). Indocyanine green-loaded polydopamine-reduced graphene oxide nanocomposites with amplifying photoacoustic and photothermal effects for cancer theranostics. *Theranostics, 6*(7), 1043. 10.7150/thno.14566 PMC4876628 27217837

[166] Chen, J., et al. (2016). Indocyanine green loaded reduced graphene oxide for in vivo photoacoustic/fluorescence dual-modality tumor imaging. *Nanoscale Research Letters, 11*, 1–11. 10.1186/s11671-016-1288-x PMC4751099 26868422

[167] Miao, X., et al. (2019). Graphene quantum dots wrapped gold nanoparticles with integrated enhancement mechanisms as sensitive and homogeneous substrates for surface-enhanced Raman spectroscopy. *Analytical Chemistry, 91*(11), 7295–7303. 10.1021/acs.analchem.9b01001 31062958

[168] Rifai, A., Pirogova, E. & Fox, K. (2019). *Diamond, carbon nanotubes and graphene for biomedical applications.*

[169] Liu, X., et al. (2020). Reduced graphene oxide/mesoporous silica nanocarriers for pH-triggered drug release and photothermal therapy. *ACS Applied Bio Materials, 3*(5), 2577–2587. 10.1021/acsabm.9b01108 35025390

[170] Yan, Z., et al. (2022). Visible-light-responsive reduced graphene oxide/gC 3 N 4/TiO 2 composite nanocoating for photoelectric stimulation of neuronal and osteoblastic differentiation. *RSC Advances, 12*(15), 8878–8888. 10.1039/d2ra00282e PMC8985170 35424887

[171] Işıklan, N., Hussien, N. A., & Türk, M. (2021). Synthesis and drug delivery performance of gelatin-decorated magnetic graphene oxide nanoplatform. *Colloids and Surfaces A: Physicochemical and Engineering Aspects, 616*, 126256.

[172] Halouane, F., et al. (2017). Selective isolation and eradication of E. coli associated with urinary tract infections using anti-fimbrial modified magnetic reduced graphene oxide nanoheaters. *Journal of Materials Chemistry B, 5*(40), 8133–8142. 10.1039/c7tb01890h 32264652

[173] Jaswal, R., et al. (2020). Nanographene enfolded AuNPs sophisticatedly synchronized polycaprolactone based electrospun nanofibre scaffold for peripheral nerve regeneration. *Materials Science and Engineering: C, 116*, 111213. 10.1016/j.msec.2020.111213 32806222

[174] Zeng, W. -N., et al. (2021). Mitochondria-targeting graphene oxide nanocomposites for fluorescence imaging-guided synergistic phototherapy of drug-resistant osteosarcoma. *Journal of Nanobiotechnology, 19*, 1–19. 10.1186/s12951-021-00831-6 PMC7980640 33740998

[175] Taheri-Kafrani, A., et al. (2021). Functionalized graphene oxide/Fe3O4 nanocomposite: A biocompatible and robust nanocarrier for targeted delivery and release of anticancer agents. *Journal of Biotechnology, 331*, 26–36. 10.1016/j.jbiotec.2021.03.005 33722630

[176] Shin, H., et al. (2021). A graphene oxide-based fluorescent nanosensor to identify antiviral agents via a drug repurposing screen. *Biosensors and Bioelectronics, 183*, 113208. 10.1016/j.bios.2021.113208 33839535

[177] Alipour, N., & Namazi, H. (2020). Chelating ZnO-dopamine on the surface of graphene oxide and its application as pH-responsive and antibacterial nanohybrid delivery agent for doxorubicin. *Materials Science and Engineering: C, 108*, 110459. 10.1016/j.msec.2019.110459 31924031

[178] Zhang, X., et al. (2017). Polydopamine-functionalized nanographene oxide: a versatile nanocarrier for chemotherapy and photothermal therapy. *Nanotechnology, 28*(29), 295102. 10.1088/1361-6528/aa761b 28656906

[179] Mousavi, A., et al. (2021). Biohybrid oxidized alginate/myocardial extracellular matrix injectable hydrogels with improved electromechanical properties for cardiac tissue engineering. *International Journal of Biological Macromolecules, 180*, 692–708. 10.1016/j.ijbiomac.2021.03.097 33753199

[180] Thirumalraj, B., et al. (2018). Highly sensitive fluorogenic sensing of L-Cysteine in live cells using gelatin-stabilized gold nanoparticles decorated graphene nanosheets. *Sensors and Actuators B: Chemical, 259*, 339–346. 10.1016/j.snb.2017.12.028 PMC7127153 32288250

[181] Zhao, H., et al. (2017). Graphene-based nanomaterials for drug and/or gene delivery, bioimaging, and tissue engineering. *Drug Discovery Today, 22*(9), 1302–1317. 10.1016/j.drudis.2017.04.002 28869820

[182] Liu, Z., et al. (2008). PEGylated nanographene oxide for delivery of water-insoluble cancer drugs. *Journal of the American Chemical Society, 130*(33), 10876–10877. 10.1021/ja803688x PMC2597374 18661992

[183] Jin, R., et al. (2013). Self-assembled graphene–dextran nanohybrid for killing drug-resistant cancer cells. *ACS Applied Materials & Interfaces, 5*(15), 7181–7189. 10.1021/am401523y 23875578

[184] Zhang, L., et al. (2010). Functional graphene oxide as a nanocarrier for controlled loading and targeted delivery of mixed anticancer drugs. *Small, 6*(4), 537–544. 10.1002/smll.200901680 20033930

[185] Dinda, S., et al. (2016). Grafting of ZnS: Mn‐doped nanocrystals and an anticancer drug onto graphene oxide for delivery and cell labeling. *ChemPlusChem, 81*(1), 100–107. 10.1002/cplu.201500349 31968740

[186] Huang, P., et al. (2015). Surface functionalization of chemically reduced graphene oxide for targeted photodynamic therapy. *Journal of Biomedical Nanotechnology, 11*(1), 117–125. 10.1166/jbn.2015.2055 PMC5218590 26301305

[187] Chow, Y. T., et al. (2016). Single cell transfection through precise microinjection with quantitatively controlled injection volumes. *Scientific Reports, 6*(1), 24127. 10.1038/srep24127 PMC4828701 27067121

[188] Chong, Z. X., Yeap, S. K. & Ho, W. Y. (2021). Transfection types, methods and strategies: a technical review. *PeerJ, 9*, e11165. 10.7717/peerj.11165 PMC8067914 33976969

[189] Sheikh, S., Coutts, A. S., & La Thangue, N. B. (2017). Transfection. In* Basic science methods for clinical researchers* (p. 191–209). Elsevier.

[190] Shi, B., et al. (2018). An improved method for increasing the efficiency of gene transfection and transduction. *International Journal of Physiology, Pathophysiology and Pharmacology, 10*(2), 95. PMC5943608 29755642

[191] Sork, H., et al. (2016). Lipid-based transfection reagents exhibit cryo-induced increase in transfection efficiency. *Molecular Therapy Nucleic Acids*, 5. 10.1038/mtna.2016.8 PMC5014464 27111416

[192] Godbey, W., Zhang, X., & Chang, F. (2008). The importance of and a method for including transfection efficiency into real‐time PCR data analyses. *Biotechnology and Bioengineering, 100*(4), 765–772. 10.1002/bit.21811 18306419

[193] Sontakke, A. D., et al. (2024). Room temperature synthesis of β-cyclodextrin functionalized graphene oxide decorated MIL-100 (Fe): A sustainable drug cargo for anticancer drug delivery. *Materials Today Communications, 38*, 108560.

[194] Wang, M., et al. (2024). Potential applications of PEI loaded graphene oxide quantum dots in safe and efficient DNA delivery. *Journal of Drug Delivery Science and Technology*, 101, 106146.

[195] Tong, L., et al. (2023). Research status and prospect of non-viral vectors based on siRNA: a review. *International Journal of Molecular Sciences, 24*(4), 3375. 10.3390/ijms24043375 PMC9962405 36834783

[196] Shin, S. R., et al. (2013). Cell‐laden microengineered and mechanically tunable hybrid hydrogels of gelatin and graphene oxide. Advanced Materials, 25(44), 6385–6391. 10.1002/adma.201301082 PMC3898458 23996513

[197] Yoon, H. H., et al. (2014). Dual roles of graphene oxide in chondrogenic differentiation of adult stem cells: cell‐adhesion substrate and growth factor‐delivery carrier. *Advanced Functional Materials, 24*(41), 6455–6464.

[198] Luong, A. H., Istiqomah, D., & Lin, W. -C. (2024) Study of mechanical property and biocompatibility of graphene oxide/MEO2MA hydrogel scaffold for wound healing application. *Biomedical Engineering Letters, 14*(3), 537–548. 10.1007/s13534-024-00349-4 PMC11026359 38645584

[199] Li, M., et al. (2024). Improved biocompatibility and osteogenesis of porous graphene oxide/silk fibroin scaffold for potential applications in bone tissue engineering. *Materials Today Communications, 40*, 109480.

[200] Hosseini, F. S., et al. (2025). Graphene oxide in bone regenerative engineering: Current challenges and future perspectives. *ACS Bio & Med Chem Au, 5*(3), 350–364. 10.1021/acsbiomedchemau.4c00152 PMC12183520 40556777

[201] Grecca, I. S. G., et al. (2025). Integrating graphene oxide and mesenchymal stem cells in 3d-printed systems for drug delivery and tissue regeneration. *Pharmaceutics, 17*(8), 1088. 10.3390/pharmaceutics17081088 PMC12389577 40871107

[202] Xi, M., et al. (2026). Graphene oxide–based biomaterials in sports medicine. *BIO Integration, 7*(1), 1–20.

[203] Sharma, H., & Mondal, S. (2020). Functionalized graphene oxide for chemotherapeutic drug delivery and cancer treatment: A promising material in nanomedicine. *International Journal of Molecular Sciences, 21*(17), 6280. 10.3390/ijms21176280 PMC7504176 32872646

[204] Buskaran, K., et al. (2021). Graphene oxide loaded with protocatechuic acid and chlorogenic acid dual drug nanodelivery system for human hepatocellular carcinoma therapeutic application. *International Journal of Molecular Sciences, 22*(11), 5786. 10.3390/ijms22115786 PMC8198262 34071389

[205] Patel, S. C., et al. (2016). Graphene-based platforms for cancer therapeutics. *Therapeutic delivery, 7*(2), 101–116. 10.4155/tde.15.93 PMC4976992 26769305

[206] Gu, Z., et al. (2019). Graphene‐based smart platforms for combined Cancer therapy. *Advanced Materials, 31*(9), 1800662. 10.1002/adma.201800662 30039878

[207] Lin, B., et al. (2019). Acidic pH and high-H2O2 dual tumor microenvironment-responsive nanocatalytic graphene oxide for cancer selective therapy and recognition. *ACS Applied Materials & Interfaces, 11*(12), 11157–11166. 10.1021/acsami.8b22487 30869853

[208] Imani, R., Mohabatpour, F. & Mostafavi, F. (2018). Graphene-based nano-carrier modifications for gene delivery applications. *Carbon, 140*, 569–591.

[209] Lo, P.-Y., et al. (2020). GFP plasmid and chemoreagent conjugated with graphene quantum dots as a novel gene delivery platform for colon cancer inhibition in vitro and in vivo. *ACS Applied Bio Materials, 3*(9), 5948–5956. 10.1021/acsabm.0c00631 35021823

[210] Žárská, L., et al. (2024). Dual drug delivery in cancer therapy using graphene oxide-based nanoplatforms. *Advanced NanoBiomed Research, 4*(9), 2400026.

[211] Wu, Y., et al. (2026). Revolutionizing cancer treatment: The multifaceted role of graphene oxide in modern oncology. *Bioengineering & Translational Medicine, 11*(1), e70055. 10.1002/btm2.70055 PMC12821228 41573372

[212] Zhan, M., et al. (2025). Graphene oxide research: Current developments and future directions. *Nanomaterials, 15*(7), 507. 10.3390/nano15070507 PMC11990175 40214552

[213] Abdelhamid, H. N., & Hussein, K. H. (2021) Graphene oxide as a carrier for drug delivery of methotrexate. *Biointerface Research in Applied Chemistry, 11*(6), 14726–14735.

[214] Hashemzadeh, H., & Raissi, H. (2020). Understanding loading, diffusion and releasing of doxorubicin and paclitaxel dual delivery in graphene and graphene oxide carriers as highly efficient drug delivery systems. *Applied Surface Science, 500*, 144220.

[215] Muazim, K., & Hussain, Z. (2017). Graphene oxide—A platform towards theranostics. *Materials Science and Engineering: C, 76*, 1274–1288. 10.1016/j.msec.2017.02.121 28482495

[216] Schilcher, K., & Horswill, A. R. (2020). Staphylococcal biofilm development: structure, regulation, and treatment strategies. *Microbiology and Molecular Biology Reviews, 84*(3). 10.1128/mmbr.00026-19 PMC7430342 32792334

[217] Mohammed, H., et al. (2020). Antimicrobial mechanisms and effectiveness of graphene and graphene-functionalized biomaterials. A scope review. *Frontiers in Bioengineering and Biotechnology, 8*, 465. 10.3389/fbioe.2020.00465 PMC7261933 32523939

[218] Staneva, A. D., et al. (2021). Antibiofouling activity of graphene materials and graphene-based antimicrobial coatings. *Microorganisms, 9*(9), 1839. 10.3390/microorganisms9091839 PMC8472838 34576733

[219] Pandit, S., et al. (2021). Graphene‐based antimicrobial biomedical surfaces. *ChemPhysChem, 22*(3), 250–263. 10.1002/cphc.202000769 PMC7898826 33244859

[220] Darini, R., et al. (2025). Antimicrobial properties of Graphene sheets embedded with Titanium Oxide and Calcium Oxide nanoparticles for industrial wastewater treatment. *Scientific Reports, 15*(1), 1007. 10.1038/s41598-024-84335-x PMC11704011 39762454

